# Supplementary material for: Multi-Omics Analysis of the Mechanism of Mentha Haplocalyx Briq on the Growth and Metabolic Regulation of Fattening Sheep
Source: Animals (Basel). 2023 Nov 9;13(22):3461. doi: 10.3390/ani13223461 (PMC10668852; doi:10.3390/ani13223461)
Supplement: Supplementary file 1 [file animals-13-03461-s001.zip › animals-2663351-supplementary.pdf]

Supplementary Materials

Table S1. Main components of the diet for fattening sheep during the experimental period.

Item	CON	MHB
Ingredient（%）		
Ground corn grain	28.00	25.76
Soybean meal	15.00	13.80
Rapeseed Meal	9.00	8.28
Wheat bran	4.00	3.68
Sodium bicarbonate	1.00	0.92
Salt	1.00	0.92
Dicalcium phosphate	0.50	0.46
Calcium carbonate	0.50	0.46
Premix [1]	1.00	0.92
Mentha haplocalyx Briq.	0.00	8.00
Peanut straw	15.00	13.80
Soybean straw	25.00	23.00
Chemical composition（%）		
CP	15.10	14.28
NDF	38.70	39.27
ADF	23.20	24.16
Ether extract	3.10	2.97
Calcium	0.75	0.69
Phosphorus	0.43	0.39
Metabolizable energy[2]，MJ/Kg	9.83	9.72

Abbreviations: CON = control diet; MHB = mentha haplocalyx briq.

[1] Formulated to provide (per kilogram of premix) 600 KIU of Vitamin A, 80 KIU of vitamin D3, 5 000 IU of Vitamin E, 8 000 mg of Zn, 60 mg of Se, 200 mg of I, 9 400 mg of Fe, 72 mg of Co, 10400 mg of Mn, and 1 600 mg of Cu.

[2] Calculated according to Ministry of Agriculture of P.R. China, 2004.

Table S2. Classification composition of rumen level microbiota in fattening sheep.

Bacteria	MHB-1	MHB-2	MHB-3	MHB-4	MHB-5	MHB-6	CON-1	CON-2	CON-3	CON-4	CON-5	CON-6	Average	SD
Unclassified	43.05	45.24	33.37	45.39	54.18	46.89	42.86	42.09	41.43	52.01	54.28	50.63	45.95	6.098
Prevotella	32.72	27.86	34.39	24.04	19.63	29.85	33.91	28.62	31.75	26.34	20.12	29.01	28.19	4.928
Methanobrevibacter	1.76	3.49	15.25	6.79	7.72	4.83	5.45	5.9	3.75	4.01	8.04	3.34	5.86	3.505
Succiniclasticum	8.48	6.13	0.92	4.56	2.77	3.43	4.13	2.95	11.59	1.79	2.08	4.11	4.41	3.038
Clostridium IV	0.64	0.87	1.32	6.49	2.21	3.58	1.24	1.66	0.2	4.1	2.38	0.66	2.11	1.820
Selenomonas	1.86	1.26	0.59	0.88	0.88	2.03	3.31	2.71	0.12	2.07	0.49	1.1	1.44	0.962
Paraprevotella	1.43	0.51	0.81	2.1	2.45	0.7	0.35	0.97	0.43	0.65	0.99	0.86	1.02	0.657
Butyrivibrio	0.92	1.49	0.47	0.1	0.67	0.33	1.71	0.68	0.55	1.13	1.51	0.99	0.88	0.505
Barnesiella	0.46	0.81	1.17	0.6	0.21	0.36	0.37	0.9	0.03	0.78	3.29	0.98	0.83	0.844
Ruminococcus	0.41	0.49	0.12	2.81	0.25	0.2	0.63	4.72	1.25	0.24	0.74	0.3	1.01	1.383
Centipeda	2.38	0.53	0.81	0.16	0.77	0.66	0.27	1.3	0	0.74	0.12	0.25	0.67	0.655
Anaeroplasma	0.77	1.03	0.41	0.24	0.96	0.85	0.8	0.21	0.04	0.48	0.62	0.45	0.57	0.317
Acetobacteroides	0.01	1.37	2.53	0.04	0.28	0.05	0.08	2.78	0	0.35	0.02	0	0.63	1.023
Bifidobacterium	0.19	0	2.28	0	0.17	2.5	0	0.01	0.73	0.23	0.27	0	0.53	0.893
Treponema	0.31	0.57	0.39	1.58	0.27	0.72	0.21	0.66	0.9	0.11	0.55	0.23	0.54	0.404
Sporobacter	0.24	0.38	0.48	0.09	0.98	0.25	0.26	0.33	0.23	0.07	0.17	1.45	0.41	0.405
Oscillibacter	0.11	1.22	0.02	0.44	0.06	0.03	0.05	0.02	0	0.01	0.03	1.66	0.30	0.552
Saccharofermentans	0.37	0.33	0.18	0.55	0.52	0.06	0.27	0.11	0.42	0.34	0.38	0.43	0.33	0.152
Pseudobutyrivibrio	0.04	1.04	0.35	0.2	0.17	0.04	0.18	0.09	0.05	0.05	0.23	0.51	0.25	0.288
Stomatobaculum	0.47	0.16	0.1	0.12	0.19	0.42	0.1	0.13	0.5	0.12	0.21	0.1	0.22	0.153
Saccharibacteria_genera_incertae_sedis	0.56	0.37	0.21	0.07	0.43	0.06	0.23	0.16	0.03	0.05	0.11	0.08	0.20	0.172
Fibrobacter	0.41	0.1	0.15	0.02	0.25	0.02	0.49	0.48	0.01	0	0.19	0.16	0.19	0.182
Roseburia	0.04	0.04	1.22	0.01	0.18	0.03	0.06	0.02	0.03	0.5	0.01	0.01	0.18	0.356

Mogibacterium	0.06	0.12	0.25	0.08	0.23	0.2	0.26	0.15	0.19	0.15	0.2	0.14	0.17	0.064
Lachnospiracea_incertae_sedis	0.17	0.26	0.21	0.06	0.08	0.04	0.09	0.2	1.12	0.03	0.03	0.07	0.20	0.301
Schwartzia	0.19	0.24	0.09	0.11	0.07	0.24	0.12	0.37	0.25	0.04	0.16	0.11	0.17	0.095
Fretibacterium	0.01	0.1	0.01	0	0	0.01	0.06	0.01	0.01	1.39	0.35	0.08	0.17	0.397
Olsenella	0.03	0.04	0.03	0.06	0.15	0.21	0.08	0.04	1.21	0.02	0.13	0.06	0.17	0.332
Lactonifactor	0.02	0.15	0.44	0	0.19	0.01	0.08	0.12	0	0.25	0.1	0.08	0.12	0.128
Blautia	0.07	0.13	0.07	0.07	0.13	0.03	0.2	0.09	0.05	0.09	0.26	0.21	0.12	0.072
Anaerovorax	0.06	0.17	0.18	0.09	0.2	0.06	0.16	0.19	0.08	0.06	0.11	0.09	0.12	0.055
Intestinimonas	0.11	0.23	0.01	0.09	0.15	0.03	0.1	0.04	0.06	0.03	0.23	0.17	0.10	0.076
Holdemania	0.08	0.05	0.08	0.2	0.24	0.02	0.05	0.04	0.59	0.06	0.08	0.05	0.13	0.160
Pirellula	0.01	0.01	0.01	0.01	0.02	0.12	0.39	0.01	0	0.16	0.4	0.01	0.10	0.149
Alkalibacter	0.01	0.27	0.05	0.19	0.06	0.03	0.02	0.06	0	0.43	0.01	0.08	0.10	0.131
Subdivision5_genera_incertae_sedis	0.09	0.09	0.06	0.18	0.12	0.14	0.08	0.11	0	0.07	0.05	0.08	0.09	0.046
Desulfovibrio	0.05	0.11	0.06	0.03	0.02	0	0.25	0	0.01	0.11	0.01	0.22	0.07	0.085
Syntrophococcus	0.05	0.04	0.04	0.07	0.4	0.04	0.03	0.03	0.04	0.03	0.04	0.01	0.07	0.105
Moryella	0.09	0.05	0.06	0.05	0.15	0.03	0.09	0.06	0.08	0.07	0.08	0.05	0.07	0.031
Alloprevotella	0.11	0.25	0.06	0.03	0.04	0.09	0	0.01	0.04	0.01	0.05	0.06	0.06	0.067
Ornatilinea	0.01	0.06	0	0.03	0.02	0.03	0.11	0.12	0.23	0.06	0.1	0.09	0.07	0.064
Succinivibrio	0.14	0.01	0.09	0.02	0.03	0.05	0.05	0.04	0	0.01	0.05	0.11	0.05	0.043
Anaerovibrio	0.04	0.08	0.15	0.13	0.01	0.04	0.05	0.09	0	0.04	0.01	0.03	0.06	0.048
Acetatifactor	0.01	0.01	0.01	0.02	0.04	0.01	0.03	0.01	0.65	0.01	0.03	0	0.07	0.183
Desulfobulbus	0.1	0.07	0	0.01	0.06	0.03	0.05	0.08	0.07	0.06	0.03	0.03	0.05	0.030
Marinilabilia	0.01	0.13	0.01	0.01	0.19	0.07	0	0	0.04	0.01	0.02	0.01	0.04	0.060
Lachnospira	0	0	0	0	0.12	0.01	0.17	0.04	0	0	0.01	0.15	0.04	0.065
Asteroleplasma	0.03	0.18	0.02	0.02	0.03	0.01	0.01	0.03	0.01	0.01	0.14	0.02	0.04	0.056
Succinimonas	0.03	0.46	0	0	0	0	0	0	0	0	0	0	0.04	0.132

Sphaerochaeta	0.02	0	0.02	0.48	0.02	0.02	0.01	0.03	0.01	0	0.01	0.01	0.05	0.135
Bacteroides	0.01	0.08	0.01	0.03	0.01	0.03	0.08	0.04	0.2	0.04	0.01	0.02	0.05	0.054
Hydrogenoanaerobacterium	0	0.02	0	0	0.35	0	0.01	0	0	0	0	0	0.03	0.100
Lachnobacterium	0	0.2	0.01	0	0	0	0	0.02	0.06	0	0	0.12	0.03	0.064
Methanosphaera	0.04	0.05	0.04	0.06	0.04	0.01	0.03	0.02	0.06	0.03	0.02	0.02	0.04	0.016
Clostridium XlVa	0.02	0.26	0.01	0	0	0.01	0	0.01	0	0.01	0.01	0.03	0.03	0.073
Bulleidia	0	0.03	0.03	0.03	0.12	0.03	0.01	0.02	0.01	0.01	0.04	0	0.03	0.032
Anaerobacterium	0.07	0.15	0	0.02	0.01	0	0.03	0.01	0	0	0.03	0.02	0.03	0.043
Eubacterium	0.07	0.06	0.01	0.02	0.01	0.02	0.01	0	0.09	0.01	0.02	0.04	0.03	0.029
Falsiporphyromonas	0.01	0.01	0	0	0.01	0.01	0.01	0	0	0.21	0.01	0.08	0.03	0.061
Acinetobacter	0.03	0.03	0.04	0.02	0.03	0.04	0.02	0.03	0.02	0.02	0.03	0.02	0.03	0.008
Howardella	0.02	0.05	0.03	0.03	0.03	0.03	0.02	0.03	0.03	0.02	0.03	0.01	0.03	0.010
Methanomassiliicoccus	0.02	0.01	0.03	0.01	0	0.05	0.01	0.03	0	0.02	0.02	0.02	0.02	0.014
Pseudobacteroides	0.02	0.06	0.01	0	0	0	0.06	0.01	0	0.03	0	0.03	0.02	0.022
Pyramidobacter	0.03	0.02	0.01	0	0.01	0.04	0.01	0.04	0.03	0.02	0.01	0.01	0.02	0.013
Faecalibacterium	0.02	0.05	0	0.02	0.02	0.01	0.03	0.04	0.01	0.02	0	0	0.02	0.016
Dorea	0.01	0.01	0.01	0	0.02	0	0.01	0.01	0.02	0	0.05	0.04	0.02	0.016
Atopobium	0	0.01	0.01	0.01	0.04	0.01	0.01	0.01	0.03	0.01	0.02	0.01	0.01	0.011
Flavonifractor	0.06	0.01	0	0.05	0	0	0	0.02	0	0	0	0.01	0.01	0.021
Phocaeicola	0.01	0	0.01	0.02	0.07	0.01	0.01	0.01	0	0.01	0	0	0.01	0.019
Suttonella	0.03	0.04	0	0	0	0.02	0.01	0.01	0.03	0.02	0.01	0.01	0.02	0.013
Oribacterium	0.11	0	0	0	0	0	0	0	0.02	0	0	0	0.01	0.032
Vampirovibrio	0.01	0.01	0.01	0.07	0.01	0.02	0	0.01	0	0	0	0.01	0.01	0.019
Shuttleworthia	0	0.02	0	0	0	0	0.01	0	0.03	0	0	0.05	0.01	0.016
Ruminococcus2	0.05	0.01	0.01	0	0.01	0	0.01	0.01	0.01	0	0	0	0.01	0.014
Lachnoanaerobaculum	0.01	0.01	0.01	0.01	0.02	0	0	0	0.01	0.01	0.02	0.01	0.01	0.007

Eisenbergiella	0	0.01	0	0.11	0	0	0.01	0	0	0	0	0	0.01	0.031
Anaerobiospirillum	0.03	0.01	0	0	0.03	0	0	0	0	0	0.02	0.01	0.01	0.012
Anaerocella	0.02	0	0	0.01	0	0.01	0	0	0.04	0.01	0	0	0.01	0.012
Anaerosporobacter	0	0.02	0	0.01	0.02	0.01	0.01	0	0.01	0	0	0.01	0.01	0.008
Marvinbryantia	0	0.01	0	0	0	0	0	0	0.08	0	0	0.01	0.01	0.023
Coprococcus	0.01	0	0	0.01	0.01	0.01	0	0	0.04	0	0.01	0	0.01	0.011
Streptococcus	0	0.01	0.01	0.01	0	0.01	0.01	0	0	0.01	0.01	0	0.01	0.005
Campylobacter	0	0.01	0	0	0	0.03	0.01	0	0	0	0	0	0.00	0.009
Gimesia	0	0	0	0	0.01	0	0.01	0.02	0	0.01	0	0.01	0.01	0.007
Mitsuokella	0	0	0	0	0	0	0	0	0.05	0.02	0	0	0.01	0.015
Phascolarctobacterium	0	0.01	0	0	0	0	0	0	0	0.02	0.01	0	0.00	0.007
Methylobacterium	0	0	0.01	0.01	0.01	0.01	0	0	0	0	0	0	0.00	0.005
Aestuariispira	0	0.01	0	0.04	0	0	0	0.01	0	0	0	0	0.01	0.012
Brachymonas	0	0	0	0	0.01	0	0	0.01	0.01	0	0	0	0.00	0.005
Candidatus Endomicrobium	0	0.03	0	0	0	0	0	0	0	0	0	0	0.00	0.009
Denitrobacterium	0	0	0	0	0	0	0	0	0.01	0.01	0.01	0	0.00	0.005
Alistipes	0	0	0	0	0	0.01	0	0.02	0	0	0	0	0.00	0.006
Papillibacter	0	0.01	0.01	0	0	0	0	0	0	0	0	0	0.00	0.004
Guggenheimella	0	0	0	0	0	0	0	0	0.06	0	0	0	0.01	0.017
Pseudomonas	0	0	0.01	0	0	0	0	0	0.01	0	0	0	0.00	0.004
Mannheimia	0	0	0	0	0	0.01	0	0	0	0	0	0.01	0.00	0.004
Ralstonia	0	0	0	0	0	0	0	0	0.01	0	0	0	0.00	0.003
Moraxella	0	0	0.01	0	0	0	0	0	0	0	0	0	0.00	0.003
Bacillus	0	0.01	0	0	0	0	0	0	0.01	0	0	0	0.00	0.004
Rhizobium	0	0	0	0	0	0	0.01	0	0	0	0	0	0.00	0.003
Enterorhabdus	0	0	0	0	0	0	0	0	0	0	0.01	0	0.00	0.003

Acetoanaerobium	0	0.01	0	0	0	0	0	0	0	0	0	0	0.00	0.003
Brevundimonas	0	0	0	0	0	0	0	0	0.01	0	0	0	0.00	0.003
Heliobacillus	0	0	0.01	0	0	0	0	0.01	0	0.01	0	0	0.00	0.005
Acholeplasma	0	0	0	0	0	0.02	0	0	0	0	0	0	0.00	0.006
Acidaminococcus	0	0	0	0	0	0	0	0	0.03	0	0	0	0.00	0.009
Aggregicoccus	0.01	0	0	0	0	0	0	0	0	0	0	0	0.00	0.003
Aureimonas	0	0	0	0.01	0	0	0	0.01	0	0	0	0	0.00	0.004
Paraeggerthella	0	0	0	0	0	0	0	0	0	0	0	0	0.00	0.000
Parvibacter	0	0	0.01	0	0	0	0	0	0	0	0	0	0.00	0.003
Pseudoramibacter	0	0	0	0	0	0	0	0	0.03	0	0	0	0.00	0.009
Anaerofilum	0.01	0	0	0	0	0	0	0	0	0	0	0	0.00	0.003
Candidatus Brocadia	0.01	0	0.01	0	0	0	0	0	0	0	0	0	0.00	0.004
SR1_genera_incertae_sedis	0.01	0	0	0	0	0	0	0	0	0	0	0	0.00	0.003
Defluviitalea	0	0	0.01	0	0	0	0	0	0	0	0	0	0.00	0.003
Escherichia/Shigella	0	0	0	0	0	0	0	0	0.01	0.01	0	0	0.00	0.004
Alysiella	0	0	0	0	0	0	0	0	0	0	0	0	0.00	0.000
Dialister	0	0	0	0	0	0	0	0	0.02	0	0	0	0.00	0.006
Gemmobacter	0	0	0	0	0	0	0	0	0	0	0	0	0.00	0.000
Slackia	0	0	0	0	0	0	0	0	0.01	0	0	0	0.00	0.003
Fusobacterium	0	0	0	0	0	0	0	0	0	0	0	0	0.00	0.000
Devosia	0	0	0	0	0	0	0	0	0	0	0	0	0.00	0.000
Senegalimassilia	0	0	0	0	0	0	0	0	0	0	0	0	0.00	0.000
Lysinibacillus	0	0	0	0	0	0	0	0	0	0	0	0	0.00	0.000
Ruminobacter	0	0	0	0	0	0	0	0	0	0	0	0.01	0.00	0.003
Enterobacter	0	0	0	0	0	0	0	0	0	0	0	0	0.00	0.000
Kandleria	0	0	0	0	0	0	0	0	0	0	0	0	0.00	0.000

Bradyrhizobium	0	0	0	0	0	0	0	0	0.01	0	0	0	0.00	0.003
Eikenella	0	0	0	0	0	0	0	0	0	0	0	0	0.00	0.000
Porphyromonas	0	0	0	0	0	0	0	0	0	0	0	0	0.00	0.000
Sphingomonas	0	0	0	0	0	0	0	0	0	0	0	0	0.00	0.000
Acetivibrio	0	0	0	0	0	0	0	0.01	0	0	0	0	0.00	0.003
Clostridium XlVb	0	0	0	0	0	0	0	0	0	0	0	0	0.00	0.000
Hespellia	0	0	0	0	0	0	0	0	0.01	0	0	0	0.00	0.003
Anaerophaga	0	0	0	0.01	0	0	0	0	0	0	0	0	0.00	0.003
Parabacteroides	0	0	0	0	0	0	0	0	0	0	0	0	0.00	0.000
Hungatella	0	0	0	0	0	0	0	0	0	0	0	0	0.00	0.000
Prauserella	0	0	0	0	0	0	0	0	0	0	0	0	0.00	0.000
Terribacillus	0	0	0	0	0	0	0	0	0	0	0	0	0.00	0.000
Peptococcus	0	0	0	0	0	0	0	0	0.01	0	0	0	0.00	0.003
Paenibacillus	0	0	0	0	0	0	0	0	0	0	0	0	0.00	0.000
Spirochaeta	0	0	0	0	0	0	0	0	0	0	0	0	0.00	0.000
Vallitalea	0	0	0	0	0	0	0	0	0	0	0	0	0.00	0.000
Anaerofustis	0	0	0	0	0	0	0	0	0	0	0	0	0.00	0.000
Murimonas	0	0	0	0	0	0	0	0	0	0	0	0	0.00	0.000
Parasutterella	0	0	0	0	0	0	0	0	0.01	0	0	0	0.00	0.003
Thermoactinomyces	0	0	0	0	0	0	0	0	0	0	0	0	0.00	0.000
Brucella	0	0	0	0	0	0	0	0	0	0	0	0	0.00	0.000
Weissella	0	0	0	0	0	0	0	0	0	0	0	0	0.00	0.000
Elizabethkingia	0	0	0	0	0	0	0	0	0	0	0	0	0.00	0.000
Bordetella	0	0	0	0	0	0	0	0	0	0	0	0	0.00	0.000
Labrenzia	0	0	0	0	0	0	0	0	0	0	0	0	0.00	0.000
Streptomyces	0	0	0	0	0	0	0	0	0	0	0	0	0.00	0.000

Actinomyces	0	0	0	0	0	0	0	0	0	0	0	0	0.00	0.000
Clostridium III	0	0	0	0	0	0	0	0	0	0	0	0	0.00	0.000
Actinotignum	0	0	0	0	0	0	0	0	0	0	0	0	0.00	0.000
Bosea	0	0	0	0	0	0	0	0	0	0	0	0	0.00	0.000
Corynebacterium	0	0	0	0	0	0	0	0	0	0	0	0	0.00	0.000
Dechloromonas	0	0	0	0	0	0	0	0	0	0	0	0	0.00	0.000
Methanobacterium	0	0	0	0	0	0	0	0	0	0	0	0	0.00	0.000
Armatimonadetes_gp2	0	0	0	0	0	0	0	0	0	0	0	0	0.00	0.000
Anaerostipes	0	0	0	0	0	0	0	0	0	0	0	0	0.00	0.000
Synergistes	0	0	0	0	0	0	0	0	0	0	0	0	0.00	0.000
Arthrobacter	0	0	0	0	0	0	0	0	0	0	0	0	0.00	0.000
Luteimonas	0	0	0	0	0	0	0	0	0	0	0	0	0.00	0.000
Laceyella	0	0	0	0	0	0	0	0	0	0	0	0	0.00	0.000
Burkholderia	0	0	0	0	0	0	0	0	0	0	0	0	0.00	0.000
Streptobacillus	0	0	0	0	0	0	0	0	0	0	0	0	0.00	0.000
Desulfuromonas	0	0	0	0	0	0	0	0	0	0	0	0	0.00	0.000
Pseudoscardovia	0	0	0	0	0	0	0	0	0	0	0	0	0.00	0.000
Cellulosimicrobium	0	0	0	0	0	0	0	0	0	0	0	0	0.00	0.000
Acidovorax	0	0	0	0	0	0	0	0	0	0	0	0	0.00	0.000
Franconibacter	0	0	0	0	0	0	0	0	0	0	0	0	0.00	0.000
Planifilum	0	0	0	0	0	0	0	0	0	0	0	0	0.00	0.000
Actinopolymorpha	0	0	0	0	0	0	0	0	0	0	0	0	0.00	0.000
Ramlibacter	0	0	0	0	0	0	0	0	0	0	0	0	0.00	0.000
Phenylobacterium	0	0	0	0	0	0	0	0	0	0	0	0	0.00	0.000
Saccharopolyspora	0	0	0	0	0	0	0	0	0	0	0	0	0.00	0.000
Angiococcus	0	0	0	0	0	0	0	0	0	0	0	0	0.00	0.000

Thermomonospora	0	0	0	0	0	0	0	0	0	0	0	0	0.00	0.000
Ureibacillus	0	0	0	0	0	0	0	0	0	0	0	0	0.00	0.000
Oceanobacillus	0	0	0	0	0	0	0	0	0	0	0	0	0.00	0.000
Microbacterium	0	0	0	0	0	0	0	0	0	0	0	0	0.00	0.000
Rhodopseudomonas	0	0	0	0	0	0	0	0	0	0	0	0	0.00	0.000
Mangrovibacterium	0	0	0	0	0	0	0	0	0	0	0	0	0.00	0.000
Faecalicoccus	0	0	0	0	0	0	0	0	0	0	0	0	0.00	0.000
Clostridium XI	0	0	0	0	0	0	0	0	0	0	0	0	0.00	0.000
Clostridium XVIII	0	0	0	0	0	0	0	0	0	0	0	0	0.00	0.000
Microvirga	0	0	0	0	0	0	0	0	0	0	0	0	0.00	0.000
Paracoccus	0	0	0	0	0	0	0	0	0	0	0	0	0.00	0.000
Aquisphaera	0	0	0	0	0	0	0	0	0	0	0	0	0.00	0.000
Mycobacterium	0	0	0	0	0	0	0	0	0	0	0	0	0.00	0.000
Ethanoligenens	0	0	0	0	0	0	0	0	0	0	0	0	0.00	0.000
Stenotrophomonas	0	0	0	0	0	0	0	0	0	0	0	0	0.00	0.000
Faecalitalea	0	0	0	0	0	0	0	0	0	0	0	0	0.00	0.000
Rothia	0	0	0	0	0	0	0	0	0	0	0	0	0.00	0.000
Johnsonella	0	0	0	0	0	0	0	0	0	0	0	0	0.00	0.000
Pseudoxanthomonas	0	0	0	0	0	0	0	0	0	0	0	0	0.00	0.000
Coprobacter	0	0	0	0	0	0	0	0	0	0	0	0	0.00	0.000
Cardiobacterium	0	0	0	0	0	0	0	0	0	0	0	0	0.00	0.000
Staphylococcus	0	0	0	0	0	0	0	0	0	0	0	0	0.00	0.000
Aquamicrobium	0	0	0	0	0	0	0	0	0	0	0	0	0.00	0.000
Sutterella	0	0	0	0	0	0	0	0	0	0	0	0	0.00	0.000
Defluviimonas	0	0	0	0	0	0	0	0	0	0	0	0	0.00	0.000
Hydrogenibacillus	0	0	0	0	0	0	0	0	0	0	0	0	0.00	0.000

Mucinivorans	0	0	0	0	0	0	0	0	0	0	0	0	0.00	0.000
Comamonas	0	0	0	0	0	0	0	0	0	0	0	0	0.00	0.000
Halomonas	0	0	0	0	0	0	0	0	0	0	0	0	0.00	0.000
Thermobacillus	0	0	0	0	0	0	0	0	0	0	0	0	0.00	0.000
Elusimicrobium	0	0	0	0	0	0	0	0	0	0	0	0	0.00	0.000
Epilithonimonas	0	0	0	0	0	0	0	0	0	0	0	0	0.00	0.000
Thermobifida	0	0	0	0	0	0	0	0	0	0	0	0	0.00	0.000
Ancylobacter	0	0	0	0	0	0	0	0	0	0	0	0	0.00	0.000
Massilia	0	0	0	0	0	0	0	0	0	0	0	0	0.00	0.000
Turicibacter	0	0	0	0	0	0	0	0	0	0	0	0	0.00	0.000
Pseudochrobactrum	0	0	0	0	0	0	0	0	0	0	0	0	0.00	0.000
Sphingopyxis	0	0	0	0	0	0	0	0	0	0	0	0	0.00	0.000
Nocardiopsis	0	0	0	0	0	0	0	0	0	0	0	0	0.00	0.000
Methyloversatilis	0	0	0	0	0	0	0	0	0	0	0	0	0.00	0.000
Pseudochelatococcus	0	0	0	0	0	0	0	0	0	0	0	0	0.00	0.000
Methylocystis	0	0	0	0	0	0	0	0	0	0	0	0	0.00	0.000
Megasphaera	0	0	0	0	0	0	0	0	0	0	0	0	0.00	0.000
Anaerotruncus	0	0	0	0	0	0	0	0	0	0	0	0	0.00	0.000
Brevibacillus	0	0	0	0	0	0	0	0	0	0	0	0	0.00	0.000
Cohnella	0	0	0	0	0	0	0	0	0	0	0	0	0.00	0.000
Ureaplasma	0	0	0	0	0	0	0	0	0	0	0	0	0.00	0.000
Candidatus Kuenenia	0	0	0	0	0	0	0	0	0	0	0	0	0.00	0.000
Catonella	0	0	0	0	0	0	0	0	0	0	0	0	0.00	0.000
Gemmiger	0	0	0	0	0	0	0	0	0	0	0	0	0.00	0.000
Pleomorphomonas	0	0	0	0	0	0	0	0	0	0	0	0	0.00	0.000
Gp6	0	0	0	0	0	0	0	0	0	0	0	0	0.00	0.000

Roseomonas	0	0	0	0	0	0	0	0	0	0	0	0	0.00	0.000
Povalibacter	0	0	0	0	0	0	0	0	0	0	0	0	0.00	0.000
Proteiniphilum	0	0	0	0	0	0	0	0	0	0	0	0	0.00	0.000
Pelagibacterium	0	0	0	0	0	0	0	0	0	0	0	0	0.00	0.000
Thermobispora	0	0	0	0	0	0	0	0	0	0	0	0	0.00	0.000
Yersinia	0	0	0	0	0	0	0	0	0	0	0	0	0.00	0.000
Rhodopirellula	0	0	0	0	0	0	0	0	0	0	0	0	0.00	0.000
Novosphingobium	0	0	0	0	0	0	0	0	0	0	0	0	0.00	0.000
Geobacter	0	0	0	0	0	0	0	0	0	0	0	0	0.00	0.000
Mesorhizobium	0	0	0	0	0	0	0	0	0	0	0	0	0.00	0.000
Fusicatenibacter	0	0	0	0	0	0	0	0	0	0	0	0	0.00	0.000
Butyricicoccus	0	0	0	0	0	0	0	0	0	0	0	0	0.00	0.000
Granulicatella	0	0	0	0	0	0	0	0	0	0	0	0	0.00	0.000
Anaerobacter	0	0	0	0	0	0	0	0	0	0	0	0	0.00	0.000
Pigmentiphaga	0	0	0	0	0	0	0	0	0	0	0	0	0.00	0.000
Conexibacter	0	0	0	0	0	0	0	0	0	0	0	0	0.00	0.000
Polymorphobacter	0	0	0	0	0	0	0	0	0	0	0	0	0.00	0.000
Gilvimarinus	0	0	0	0	0	0	0	0	0	0	0	0	0.00	0.000
Lautropia	0	0	0	0	0	0	0	0	0	0	0	0	0.00	0.000
Blastopirellula	0	0	0	0	0	0	0	0	0	0	0	0	0.00	0.000
Sphingobacterium	0	0	0	0	0	0	0	0	0	0	0	0	0.00	0.000
Lutispora	0	0	0	0	0	0	0	0	0	0	0	0	0.00	0.000
Pectinatus	0	0	0	0	0	0	0	0	0	0	0	0	0.00	0.000
Allisonella	0	0	0	0	0	0	0	0	0	0	0	0	0.00	0.000
Chlamydophila	0	0	0	0	0	0	0	0	0	0	0	0	0.00	0.000
Peptostreptococcus	0	0	0	0	0	0	0	0	0	0	0	0	0.00	0.000

Hyphomicrobium	0	0	0	0	0	0	0	0	0	0	0	0	0.00	0.000
Simplicispira	0	0	0	0	0	0	0	0	0	0	0	0	0.00	0.000
Macellibacteroides	0	0	0	0	0	0	0	0	0	0	0	0	0.00	0.000

Table S3. Classification and composition of fecal microbiota in fattening sheep.

Bacteria	MHB-1	MHB-2	MHB-3	MHB-4	MHB-5	MHB-6	CON-1	CON-2	CON-3	CON-4	CON-5	CON-6	Average	SD
unclassified	40.94	45.4	45.84	36.91	43.49	47.65	41.69	53.99	46.91	45.66	37.49	46.48	44.371	4.688
Sporobacter	12.57	10.05	7.49	7.04	5.77	7.28	14.18	8.95	8.34	5.97	10.98	8.14	8.897	2.600
Bacteroides	8.24	6.16	10.54	1.77	6.74	8.53	8.58	4.48	8.81	6.78	7.05	6.44	7.010	2.277
Treponema	3.14	2.78	3.46	11.88	8.45	3.03	2.86	2.12	4.8	1.36	3.58	2.9	4.197	2.996
Clostridium XlVa	4.24	2.36	4.31	3.94	5.78	5.55	3.97	2.96	2.28	6.54	2.72	4.97	4.135	1.391
Alistipes	4.34	2.63	5.44	1.63	1.9	3.13	1.77	6.95	4.72	5.23	2.35	4.54	3.719	1.722
Ruminococcus	3.28	1.68	0.82	3.74	5.1	4.56	4.47	2.13	2.45	1.13	8.17	3.28	3.401	2.035
Succinivibrio	1.07	2.52	4.38	3.84	2.33	0.53	1.09	0.33	0.39	4.55	8.92	2.13	2.673	2.480
Clostridium IV	2.18	2.14	2.59	4.12	2.65	3.17	1.28	3.96	3.37	2.67	1.72	2.29	2.678	0.853
Prevotella	4.35	7.91	1.48	5.85	1.9	2.27	0.04	1.27	0.22	2.78	1.53	4.13	2.811	2.348
Oscillibacter	1.32	1.2	1.9	1.66	2.09	1.44	0.98	1.39	1.34	2.45	0.93	2.25	1.579	0.494
Phascolarctobacterium	0.85	1.19	1.68	1.08	1.1	1.24	0.68	0.61	0.93	2.75	1.7	1.3	1.259	0.579
Methanobrevibacter	1.53	2.84	0.69	0.97	0.88	0.32	1.38	1.42	0.44	0.41	0.94	1.66	1.123	0.706
Roseburia	1.16	1.05	0.39	1.04	1.09	0.47	1.99	1.02	1.01	0.45	1.35	0.61	0.969	0.451
Blautia	0.85	0.52	0.31	0.83	0.19	0.19	1.16	0.46	1.93	0.57	1.05	0.51	0.714	0.495
Coprobacter	0.3	1.29	0.39	1.57	0.43	0.32	1.57	0.12	0.76	1.39	0.11	0.39	0.720	0.571
Coprococcus	0.43	0.58	0.31	0.62	0.22	0.31	0.76	0.42	0.91	0.42	1.2	0.49	0.556	0.282
Akkermansia	0.07	0.01	0.27	0	0.03	1.86	0	2.24	0	1.23	0.01	1.81	0.628	0.885
Faecalibacterium	1.16	0.04	0.13	0.77	0.16	0.47	0.75	0.18	0.78	0.11	0.97	0.31	0.486	0.384
Macellibacteroides	1.01	0.4	0.43	0.53	0.25	0	1.15	0.17	0.93	0.25	0.64	0.01	0.481	0.384
Campylobacter	0.15	0.03	0.9	1.6	0.19	0.08	0.02	0.08	0.53	0.44	0.02	0.31	0.363	0.471
Parabacteroides	0.18	0.25	0.28	0.11	0.64	0.05	0.34	0.3	0.85	0.27	0.81	0.15	0.353	0.267
Lachnospiracea_incertae_sedis	0.31	0.4	0.14	0.3	0.45	1.79	0.45	0.22	0.25	0.16	0.27	0.1	0.403	0.451
Barnesiella	0.31	0.18	0	0.31	0.25	0	1.07	0.06	0.55	0.18	0.29	0.4	0.300	0.291

Dorea	0.48	0.22	0.15	0.33	0.24	0.4	0.28	0.16	0.56	0.22	0.48	0.15	0.306	0.143
Intestinimonas	0.33	0.57	0.44	0.17	0.27	0.3	0.21	0.38	0.13	0.16	0.32	0.46	0.312	0.134
Paraprevotella	0.07	1.08	0	0.07	3.02	0	0.01	0.03	0.08	0.01	0.02	0.03	0.368	0.888
Anaerostipes	0.32	0.23	0.25	0.36	0.09	0.12	1.02	0.06	0.4	0.15	0.13	0.07	0.267	0.264
Clostridium XlVb	0.22	0.25	0.54	0.19	0.36	0.36	0.19	0.34	0.23	0.17	0.11	0.18	0.262	0.118
Papillibacter	0.26	0.4	0.12	0.21	0.39	0.16	0.18	0.53	0.14	0.24	0.25	0.28	0.263	0.122
Anaerophaga	0.23	0	0	0.84	0	0	0	0	0.59	0	0.81	0	0.206	0.338
Alloprevotella	0.01	0.59	0.03	0.92	0.11	0.01	0.11	0.01	0.42	0.27	0.27	0.1	0.238	0.282
Clostridium XVIII	0.27	0.31	0.37	0.22	0.15	0.05	0.56	0.24	0.08	0.18	0.2	0.1	0.228	0.141
Flavonifractor	0.12	0.25	0.23	0.08	0.37	0.36	0.5	0.11	0.16	0.06	0.24	0.1	0.215	0.137
Clostridium sensu stricto	0.02	0.02	0.08	0.91	0.01	0.01	1.02	0	0.06	0.08	0.03	0.03	0.189	0.364
Anaerovibrio	0.13	0.05	0.37	0.22	0	0.09	0	0.15	0.29	0.42	0.23	0.35	0.192	0.145
Kandleria	0.17	0.18	0.13	0.08	0.04	0.69	0.16	0.09	0.29	0.02	0	0.07	0.160	0.185
Anaeroplasma	0.19	0.12	0.06	0.02	0.06	0.64	0.2	0	0.03	0.08	0.32	0.01	0.144	0.183
Ruminococcus2	0.09	0.09	0.18	0.07	0.13	0.22	0.04	0.09	0.29	0.1	0.05	0.03	0.115	0.078
Parasutterella	0.11	0.07	0.07	0.09	0.06	0.01	0.15	0.06	0.18	0.25	0.14	0.11	0.108	0.064
Hungatella	0.03	0.07	0.02	0.1	0.43	0.03	0.11	0.04	0.25	0.08	0.02	0.13	0.109	0.120
Pseudoflavonifractor	0.14	0.14	0.09	0.05	0.07	0.22	0.09	0.08	0.03	0.18	0.09	0.16	0.112	0.056
Anaerotruncus	0.14	0.08	0.2	0.1	0.01	0.17	0.03	0.14	0.01	0.23	0.06	0.11	0.107	0.072
Fibrobacter	0.18	0	0	0.75	0.04	0.03	0	0.03	0.09	0	0	0	0.093	0.214
Desulfovibrio	0	0.12	0.02	0.13	0.1	0.05	0	0.03	0.15	0.29	0.12	0.15	0.097	0.083
Porphyromonas	0	0	0.1	0.09	0.03	0.06	0.12	0	0.32	0.23	0.03	0.06	0.087	0.098
Subdivision5_genera_incertae_sedis	0.03	0.18	0.54	0.03	0.03	0.08	0.03	0.06	0	0.08	0.02	0.02	0.092	0.149
Anaerobacterium	0.17	0	0	0.24	0.06	0.04	0.08	0.21	0.1	0.03	0.01	0.08	0.085	0.081
Erysipelotrichaceae_incertae_sedis	0.07	0.03	0.17	0.05	0.06	0.01	0.12	0.05	0.01	0.12	0.15	0.03	0.073	0.054
Clostridium III	0.09	0.08	0.05	0.04	0.07	0.2	0.08	0.05	0.03	0.31	0.01	0.03	0.087	0.085

Escherichia/Shigella	0.06	0.01	0	0	0	0.01	0.69	0	0.01	0.02	0.01	0	0.068	0.197
Mycoplasma	0.11	0.08	0.05	0	0.03	0.05	0.34	0.09	0.09	0.02	0.01	0.03	0.075	0.091
Butyrivibrio	0.11	0.08	0	0.02	0.33	0	0.18	0.01	0.02	0.13	0	0.07	0.079	0.099
Elusimicrobium	0.01	0.04	0.01	0.31	0.15	0.17	0	0	0.02	0.01	0	0.15	0.073	0.100
Butyricicoccus	0.08	0.04	0.08	0.07	0.04	0.08	0.07	0.05	0.08	0.11	0.07	0.04	0.068	0.021
Bifidobacterium	0.44	0	0.1	0	0.01	0.1	0	0	0	0.19	0.02	0	0.072	0.131
Acetanaerobacterium	0.1	0.08	0.09	0.03	0.07	0.04	0.03	0.09	0.04	0.18	0.02	0.03	0.067	0.046
Mogibacterium	0.07	0.08	0.04	0.07	0.02	0.02	0.05	0.12	0.03	0.09	0.04	0.03	0.055	0.031
Saccharibacteria_genera_incertae_sedis	0.04	0	0.15	0.05	0.06	0.01	0.03	0.11	0.02	0.03	0.04	0.05	0.049	0.042
Lactonifactor	0	0.05	0	0.01	0.01	0	0.11	0	0.01	0.01	0.02	0.42	0.053	0.120
Butyricimonas	0.05	0.04	0.01	0.02	0.1	0.01	0.02	0.02	0.03	0.15	0.11	0.06	0.052	0.045
Ureaplasma	0.02	0	0.06	0.01	0.12	0.01	0.05	0.03	0.1	0.02	0.07	0.03	0.043	0.038
Ethanoligenens	0.09	0.06	0	0.04	0.11	0.04	0	0.07	0.05	0	0	0.11	0.048	0.042
Guggenheimella	0	0.03	0.04	0.05	0.02	0	0.1	0.12	0.05	0.04	0.01	0.01	0.039	0.038
Holdemania	0.02	0.04	0.06	0.02	0.02	0.05	0.08	0.03	0.03	0.01	0.04	0.02	0.035	0.020
Anaerovorax	0.02	0.05	0.06	0.04	0.03	0.09	0.01	0.04	0.02	0.05	0.01	0.04	0.038	0.023
Olsenella	0.02	0.02	0	0.03	0	0.05	0.02	0.02	0.09	0.1	0.02	0	0.031	0.033
Syntrophococcus	0.04	0.02	0.05	0.07	0.01	0.02	0.03	0.03	0	0.05	0.02	0.02	0.030	0.020
Lysinibacillus	0.22	0	0.02	0.01	0.01	0	0.04	0.02	0.01	0.01	0.01	0	0.029	0.061
Anaerococcus	0.01	0.01	0	0.03	0.01	0.05	0.06	0.02	0.05	0.06	0.01	0.01	0.027	0.022
Helicobacter	0	0.06	0	0	0.2	0.01	0	0	0.09	0	0	0	0.030	0.061
Anaerofilum	0	0.01	0.03	0.04	0.01	0.01	0.02	0.02	0.01	0.09	0.06	0	0.025	0.027
Coprobacillus	0.13	0.03	0	0.02	0	0	0	0	0.03	0	0.07	0.01	0.024	0.039
Pseudobacteroides	0	0	0.23	0	0	0.02	0	0	0	0.02	0	0.01	0.023	0.066
Methanosphaera	0.03	0.06	0.02	0.02	0.01	0.02	0.02	0.04	0.01	0.04	0.03	0.02	0.027	0.014
Eisenbergiella	0.01	0	0.01	0.02	0.01	0.17	0.02	0	0.07	0.01	0	0	0.027	0.049

Acinetobacter	0.03	0.02	0.01	0.02	0	0.02	0.03	0.03	0.03	0.02	0.03	0.01	0.021	0.010
Defluviitalea	0.03	0.01	0.02	0	0.04	0.06	0.01	0.01	0.04	0.02	0.03	0.01	0.023	0.017
Subdoligranulum	0.08	0.04	0.03	0.01	0.01	0	0.03	0.02	0.01	0.02	0.01	0.02	0.023	0.021
Odoribacter	0.04	0	0.08	0.01	0.01	0	0.02	0	0.01	0.03	0.02	0	0.018	0.023
Faecalicoccus	0.06	0	0.04	0	0	0.02	0	0.02	0.02	0.02	0.02	0.03	0.019	0.018
Hespellia	0	0	0	0.12	0.03	0	0.02	0	0.03	0	0	0	0.017	0.035
Faecalitalea	0.05	0.04	0.02	0	0.01	0.02	0.02	0.02	0	0	0.04	0.01	0.019	0.017
Eubacterium	0.01	0.01	0.01	0.06	0	0.04	0.02	0.01	0.01	0.02	0.01	0.01	0.018	0.017
Arcanobacterium	0	0	0.01	0	0	0.01	0.02	0	0.11	0.01	0.01	0	0.014	0.031
Vampirovibrio	0.01	0	0.04	0.04	0.01	0.02	0	0	0	0.01	0	0.05	0.015	0.018
Peptoniphilus	0	0	0.01	0.02	0	0.01	0.06	0	0.02	0.01	0	0.02	0.013	0.017
Ruminobacter	0	0	0	0	0	0	0	0	0	0.18	0	0	0.015	0.052
Schwartzia	0	0	0	0	0.04	0	0	0	0	0.13	0	0	0.014	0.038
Lawsonia	0	0	0.06	0	0.02	0	0	0	0	0	0	0.07	0.013	0.025
Turicibacter	0	0.01	0	0	0	0	0.05	0	0	0.01	0.06	0	0.011	0.021
Streptococcus	0.02	0.01	0.02	0	0	0.01	0.04	0	0	0	0.02	0	0.010	0.013
Peptococcus	0	0	0	0	0	0	0.01	0.01	0.01	0.09	0.01	0	0.011	0.025
Fusicatenibacter	0.01	0	0.02	0	0	0.04	0.03	0	0	0	0.01	0.01	0.010	0.013
Gracilibacter	0	0	0.01	0.01	0.02	0.06	0	0.02	0	0	0	0	0.010	0.018
Hydrogenoanaerobacterium	0.01	0.03	0.01	0.01	0.01	0.02	0	0	0.01	0	0	0.02	0.010	0.010
Anaerosporobacter	0.01	0	0.02	0.01	0	0.02	0.01	0	0.01	0	0.01	0.01	0.008	0.007
Parvibacter	0.02	0	0.01	0.02	0	0.01	0	0	0.02	0	0	0.01	0.008	0.009
Negativicoccus	0	0	0	0	0	0	0.03	0	0.02	0.03	0	0.01	0.008	0.012
Aestuariispira	0	0.01	0.01	0	0	0.04	0.01	0.03	0	0	0	0	0.008	0.013
Paraeggerthella	0.01	0.02	0.01	0	0	0	0.01	0.01	0	0	0.01	0.01	0.007	0.007
Murdochiella	0	0	0	0	0	0	0.02	0.01	0.02	0.01	0.01	0	0.006	0.008

Enterococcus	0	0	0	0.07	0	0	0	0	0	0	0	0	0.006	0.020
Comamonas	0	0	0	0	0	0	0	0	0.04	0.02	0	0	0.005	0.012
Murimonas	0	0.03	0	0.01	0	0	0	0.01	0.01	0	0	0.01	0.006	0.009
Gallicola	0	0	0	0.01	0.01	0	0.02	0	0.01	0	0	0	0.004	0.007
Acetivibrio	0	0	0	0.01	0.02	0	0	0	0	0.01	0.01	0	0.004	0.007
Finegoldia	0	0	0	0	0	0.01	0.01	0	0.01	0.01	0	0	0.003	0.005
Aerococcus	0	0	0	0	0	0	0.01	0	0	0.03	0	0	0.003	0.009
Bacillus	0	0	0	0	0	0	0.01	0	0	0	0.01	0	0.002	0.004
Trueperella	0	0	0	0	0	0	0	0	0	0.03	0	0	0.003	0.009
Mobiluncus	0	0	0	0	0	0	0	0	0.03	0	0	0	0.003	0.009
Corynebacterium	0	0	0	0	0	0	0	0	0.02	0	0	0	0.002	0.006
Collinsella	0	0	0	0	0	0	0.01	0	0.01	0	0.01	0	0.003	0.005
Succiniclasticum	0	0	0	0	0	0	0	0.01	0.01	0.01	0	0.01	0.003	0.005
Erysipelothrix	0	0	0	0	0	0.01	0	0.01	0	0	0	0	0.002	0.004
Saccharofermentans	0	0	0	0.01	0	0	0	0	0	0.01	0	0	0.002	0.004
Allobaculum	0	0	0	0.03	0	0	0	0	0	0	0	0	0.003	0.009
Asteroleplasma	0.01	0	0	0	0.01	0	0	0	0	0	0.01	0	0.003	0.005
Selenomonas	0	0	0	0	0	0	0	0	0	0	0	0.01	0.001	0.003
Pseudomonas	0	0	0	0	0	0	0	0	0	0	0	0	0.000	0.000
Clostridium XI	0	0	0	0	0	0	0	0	0	0	0	0.01	0.001	0.003
Veillonella	0	0	0.01	0	0	0	0	0	0	0.01	0	0	0.002	0.004
Pseudobutyrivibrio	0	0.01	0	0	0	0	0	0	0	0	0	0	0.001	0.003
Bilophila	0	0	0	0	0.01	0	0	0	0	0.01	0	0	0.002	0.004
Stomatobaculum	0	0	0	0	0	0	0	0	0	0	0	0	0.000	0.000
Brevundimonas	0	0	0	0	0	0	0	0	0	0	0	0	0.000	0.000
Bulleidia	0	0	0	0	0	0	0	0	0	0	0	0	0.000	0.000

Falsiporphyromonas	0	0	0	0	0	0	0	0	0	0	0	0	0.000	0.000
Methanomassiliicoccus	0	0	0	0	0	0	0	0	0	0	0	0	0.000	0.000
Paenibacillus	0.01	0	0	0	0	0	0	0	0	0	0	0	0.001	0.003
Neorhizobium	0	0	0	0	0	0	0	0	0	0	0	0	0.000	0.000
Robinsoniella	0	0	0	0	0	0	0	0	0	0	0	0	0.000	0.000
Methanocorpusculum	0	0	0	0	0	0.02	0	0	0	0	0	0	0.002	0.006
Romboutsia	0	0	0	0	0	0	0	0	0	0	0	0	0.000	0.000
Flaviflexus	0	0	0	0	0	0	0	0	0.01	0	0	0	0.001	0.003
Fastidiosipila	0	0	0	0	0	0	0	0	0	0	0	0	0.000	0.000
Denitrobacterium	0	0	0	0	0	0	0	0	0	0	0	0	0.000	0.000
Lutispora	0	0	0	0	0.01	0	0	0	0	0	0	0	0.001	0.003
Aureimonas	0	0	0	0	0	0	0	0	0	0	0	0	0.000	0.000
Helcococcus	0	0	0.01	0	0	0	0	0	0	0	0	0	0.001	0.003
Streptobacillus	0	0	0.01	0	0	0	0	0	0	0	0	0	0.001	0.003
Chlamydophila	0	0	0	0	0	0	0	0	0	0	0	0	0.000	0.000
Dialister	0	0	0	0	0	0	0	0	0	0	0	0	0.000	0.000
Atopobium	0	0	0	0	0	0	0	0	0	0	0	0	0.000	0.000
Fusobacterium	0	0	0	0	0	0	0	0	0.01	0	0	0	0.001	0.003
Lachnobacterium	0	0	0	0	0	0	0	0	0	0	0	0.01	0.001	0.003
Sphaerochaeta	0	0	0	0	0	0	0	0	0	0	0	0	0.000	0.000
Acetitomaculum	0	0	0	0	0	0	0	0	0	0	0	0	0.000	0.000
Saccharopolyspora	0	0	0	0	0	0	0	0	0	0	0	0	0.000	0.000
Devosia	0	0	0	0	0	0	0	0	0	0	0	0	0.000	0.000
Anaerosphaera	0	0	0	0.01	0	0	0	0	0	0	0	0	0.001	0.003
Centipeda	0	0	0	0	0	0	0	0	0	0	0	0	0.000	0.000
Acetatifactor	0	0	0	0	0	0	0	0	0	0	0	0	0.000	0.000

Slackia	0	0	0	0	0	0	0	0	0	0	0	0	0.000	0.000
Solibacillus	0	0	0	0	0	0	0	0	0	0.01	0	0	0.001	0.003
Paracoccus	0	0	0	0	0	0	0	0	0	0	0	0	0.000	0.000
Methanobacterium	0	0	0	0	0	0	0	0	0	0	0	0	0.000	0.000
Methanoculleus	0	0	0	0	0	0	0	0	0	0	0	0	0.000	0.000
Lachnospira	0	0	0	0	0	0	0	0	0	0	0	0.01	0.001	0.003
Bradyrhizobium	0	0	0	0	0	0	0	0	0	0	0	0	0.000	0.000
Acetobacteroides	0	0	0	0	0	0	0	0	0	0	0	0	0.000	0.000
Coriobacterium	0	0	0	0	0	0	0	0	0	0	0	0	0.000	0.000
Ralstonia	0	0	0	0	0	0	0	0	0	0	0	0	0.000	0.000
Senegalimassilia	0	0	0	0	0	0	0	0	0	0	0	0	0.000	0.000
Klebsiella	0	0	0	0	0	0	0	0	0	0	0	0	0.000	0.000
Howardella	0	0	0	0	0	0	0	0	0	0	0	0	0.000	0.000
Anaerofustis	0	0	0	0	0	0	0	0	0	0	0	0	0.000	0.000
Terribacillus	0	0	0	0	0	0	0	0	0	0	0	0	0.000	0.000
Methylobacterium	0	0	0	0	0	0	0	0	0	0	0	0	0.000	0.000
Sharpea	0	0	0	0	0	0	0	0	0	0	0	0	0.000	0.000
Enterorhabdus	0	0	0	0	0	0	0	0	0	0	0	0	0.000	0.000
Methanothermobacter	0	0	0	0	0	0	0	0	0	0	0	0	0.000	0.000
Kutzneria	0	0	0	0	0	0	0	0	0	0	0	0	0.000	0.000
Sphingomonas	0	0	0	0	0	0	0	0	0	0	0	0	0.000	0.000
Ezakiella	0	0	0	0	0	0	0	0	0	0	0	0	0.000	0.000
Victivallis	0	0	0	0	0	0	0	0	0	0	0	0	0.000	0.000
Gemmobacter	0	0	0	0	0	0	0	0	0	0	0	0	0.000	0.000
Labrenzia	0	0	0	0	0	0	0	0	0	0	0	0	0.000	0.000
Pseudochelatococcus	0	0	0	0	0	0	0	0	0	0	0	0	0.000	0.000

Parasporobacterium	0	0	0	0	0	0	0	0	0	0	0	0	0.000	0.000
Aquicella	0	0	0	0	0	0	0	0	0	0	0	0	0.000	0.000
Staphylococcus	0	0	0	0	0	0	0	0	0	0	0	0	0.000	0.000
Geothermomicrobium	0	0	0	0	0	0	0	0	0	0	0	0	0.000	0.000
Weissella	0	0	0	0	0	0	0	0	0	0	0	0	0.000	0.000
Lactobacillus	0	0	0	0	0	0	0	0	0	0	0	0	0.000	0.000
Pseudoramibacter	0	0	0	0	0	0	0	0	0	0	0	0	0.000	0.000
Moryella	0	0	0	0	0	0	0	0	0	0	0	0	0.000	0.000
Brachybacterium	0	0	0	0	0	0	0	0	0	0	0	0	0.000	0.000
Arthrobacter	0	0	0	0	0	0	0	0	0	0	0	0	0.000	0.000
Ureibacillus	0	0	0	0	0	0	0	0	0	0	0	0	0.000	0.000
Ensifer	0	0	0	0	0	0	0	0	0	0	0	0	0.000	0.000
Pontibacter	0	0	0	0	0	0	0	0	0	0	0	0	0.000	0.000
Cellulomonas	0	0	0	0	0	0	0	0	0	0	0	0	0.000	0.000
Shuttleworthia	0	0	0	0	0	0	0	0	0	0	0	0	0.000	0.000
Streptomyces	0	0	0	0	0	0	0	0	0	0	0	0	0.000	0.000
Microvirga	0	0	0	0	0	0	0	0	0	0	0	0	0.000	0.000
Rhizobium	0	0	0	0	0	0	0	0	0	0	0	0	0.000	0.000
Terrisporobacter	0	0	0	0	0	0	0	0	0	0	0	0	0.000	0.000
Zoogloea	0	0	0	0	0	0	0	0	0	0	0	0	0.000	0.000
Sutterella	0	0	0	0	0	0	0	0	0	0	0	0	0.000	0.000
Plasticicumulans	0	0	0	0	0	0	0	0	0	0	0	0	0.000	0.000
Cerasicoccus	0	0	0	0	0	0	0	0	0	0	0	0	0.000	0.000
Mycobacterium	0	0	0	0	0	0	0	0	0	0	0	0	0.000	0.000
Asticcacaulis	0	0	0	0	0	0	0	0	0	0	0	0	0.000	0.000
Blastochloris	0	0	0	0	0	0	0	0	0	0	0	0	0.000	0.000

Nitrosomonas	0	0	0	0	0	0	0	0	0	0	0	0	0.000	0.000
Brevibacterium	0	0	0	0	0	0	0	0	0	0	0	0	0.000	0.000
Proteinivorax	0	0	0	0	0	0	0	0	0	0	0	0	0.000	0.000
Oligosphaera	0	0	0	0	0	0	0	0	0	0	0	0	0.000	0.000
Gemmiger	0	0	0	0	0	0	0	0	0	0	0	0	0.000	0.000
Anaerobiospirillum	0	0	0	0	0	0	0	0	0	0	0	0	0.000	0.000
Thermobifida	0	0	0	0	0	0	0	0	0	0	0	0	0.000	0.000
Sphingosinicella	0	0	0	0	0	0	0	0	0	0	0	0	0.000	0.000
Deinococcus	0	0	0	0	0	0	0	0	0	0	0	0	0.000	0.000
Aminobacter	0	0	0	0	0	0	0	0	0	0	0	0	0.000	0.000
Domibacillus	0	0	0	0	0	0	0	0	0	0	0	0	0.000	0.000
Phocaeicola	0	0	0	0	0	0	0	0	0	0	0	0	0.000	0.000
Thermoleophilum	0	0	0	0	0	0	0	0	0	0	0	0	0.000	0.000
Micrococcus	0	0	0	0	0	0	0	0	0	0	0	0	0.000	0.000
Dechloromonas	0	0	0	0	0	0	0	0	0	0	0	0	0.000	0.000
Pelagibacterium	0	0	0	0	0	0	0	0	0	0	0	0	0.000	0.000
Aquamicrobium	0	0	0	0	0	0	0	0	0	0	0	0	0.000	0.000
Marvinbryantia	0	0	0	0	0	0	0	0	0	0	0	0	0.000	0.000
Tannerella	0	0	0	0	0	0	0	0	0	0	0	0	0.000	0.000
Pyramidobacter	0	0	0	0	0	0	0	0	0	0	0	0	0.000	0.000
Candidatus Brocadia	0	0	0	0	0	0	0	0	0	0	0	0	0.000	0.000
Isosphaera	0	0	0	0	0	0	0	0	0	0	0	0	0.000	0.000
Simplicispira	0	0	0	0	0	0	0	0	0	0	0	0	0.000	0.000
Candidatus Hydrogenedens	0	0	0	0	0	0	0	0	0	0	0	0	0.000	0.000
Flavobacterium	0	0	0	0	0	0	0	0	0	0	0	0	0.000	0.000

Table S4. Significant differences in metabolites of rumen fluid between MHB and CON groups in fattening sheep.

Name	MZ[1]	R.T[2](min)	VIP[3]	*p*-Value[4]	FC[5]
**Negative**					
Phenol	93.036	38.397	1.953	0.029	1.459
5-Hydroxyhexanoic acid	131.071	190.973	1.900	0.026	1.242
Salicylic acid	137.025	38.421	2.095	0.016	1.521
3-Phenylpropanoic acid	149.062	97.849	2.315	0.016	1.567
p-Hydroxyphenylacetic acid	151.040	171.694	2.169	0.008	1.878
L-Cysteinesulfinic acid	152.002	68.174	2.122	0.014	1.322
3,4-Dihydroxybenzoate (Protocatechuic acid)	154.028	24.435	2.111	0.014	1.635
Monomethyl glutaric acid	167.034	99.352	1.782	0.021	1.560
Tropic acid	225.075	330.730	1.775	0.042	1.362
Deoxycytidine	227.091	187.353	2.487	0.001	1.538
Quinaldic acid	232.060	45.590	1.708	0.037	0.753
Thymidine	241.083	101.869	1.777	0.046	2.457
(S)-Equol	241.086	38.454	1.654	0.047	0.766
Nicotinamide	243.086	103.757	2.466	0.001	1.583
5'-Deoxyadenosine	250.094	140.405	1.819	0.025	2.264
Daidzein	253.049	48.382	2.420	0.004	0.452
cis-9-Palmitoleic acid	253.215	45.161	1.911	0.023	1.400
Primidone	255.057	26.923	1.840	0.041	1.219
2-Deoxy-D-glucose 6-phosphate	260.055	44.629	1.739	0.036	0.588
Glycitein	283.060	43.281	2.859	0.000	7.728
N4-Acetylcytidine	284.088	158.201	2.067	0.009	1.625

12-Oxo-2,3-dinor-10,15-phytodienoic acid	285.149	178.096	1.906	0.045	1.318
Diosmetin	299.054	44.096	2.809	0.000	5.562
Picrotoxinin	313.069	278. 403	1.864	0.050	1.277
4-Hydroxycinnamic acid	327.085	287.266	1.885	0.015	1.317
2-Amino-3-methoxy-benzoic acid	333.107	152.105	2.568	0.005	2.024
Phenethicillin	345.095	295.022	1.728	0.048	1.848
Camptothecin	347.110	319.794	2.461	0.027	10.014
Temazepam	359.075	45.125	1.706	0.039	0.692
Cortisol 21-acetate	403.210	44.232	1.645	0.015	1.674
Pravastatin	445.228	89.139	2.195	0.004	1.250
Oleanolic acid	455.349	41.661	2.525	0.000	8.303
Hydrocortisone succinate	499.167	100.898	1.943	0.037	2.000
Diosmin	607.161	190.419	2.825	0.004	63.088
**Positive**					
Methyl acetoacetate	99.044	43.653	1.451	0.048	1.466
Methylmalonic acid	119.035	140.575	1.774	0.011	1.974
Ala-Ala	125.071	50.228	1.462	0.031	0.403
Thymine	127.051	102.313	1.644	0.039	2.829
1,4-Dihydroxybenzene	128.071	79.520	2.466	0.004	1.938
Oxyquinoline	146.061	66.681	2.008	0.016	1.289
2-Hydroxyadenine	152.058	219.660	1.612	0.031	1.803
DL-3-Hydroxybutyric acid	168.065	311.955	2.051	0.005	0.793
Arecoline	173.132	47.812	1.748	0.030	1.247
Ethyl hydrogen malonate	174.076	101.712	1.851	0.017	1.362
(Z)-4-Decen-1-ol	179.143	178.522	1.991	0.014	1.379

Dacarbazine	183.099	111.260	2.336	0.000	1.406
2-Hydroxyphenylacetic acid	194.081	312.105	1.998	0.007	1.240
4-Methoxycinnamic acid	196.098	357.102	1.731	0.019	0.874
3-Hydroxy-L-kynurenine	207.076	52.610	1.975	0.006	1.416
Ibuprofen	207.138	178.155	2.184	0.005	1.497
Cuminaldehyde	209.117	47.882	1.529	0.039	1.639
Methyldopa	212.091	392.963	1.956	0.007	0.716
Diethyltoluamide	214.122	35.080	1.778	0.030	1.667
Ala-Ser	218.113	354.695	1.548	0.046	1.271
Val-Gln	228.131	336.220	1.833	0.012	1.693
Desoxypeganine	233.128	194.622	1.496	0.036	1.453
Nicorandil	234.052	365.345	1.387	0.023	1.803
Indoleacetic acid	236.091	345.116	2.047	0.005	1.327
N-Formylmethionine	238.070	180.435	2.154	0.001	0.617
Gly-Val	238.116	296.570	1.677	0.032	1.458
2-Dehydro-3-deoxy-D-gluconate	239.082	108.519	2.042	0.012	1.220
3-Hydroxydodecanoic acid	239.163	48.653	1.806	0.014	1.278
7,8-Dihydrobiopterin	240.108	81.820	1.991	0.007	4.966
Thymidine	243.097	102.313	1.863	0.010	2.516
Ser-Pro	244.128	302.649	2.161	0.011	2.052
Deoxyadenosine	252.111	140.909	1.683	0.019	2.127
Daidzein	255.065	49.382	2.241	0.013	0.317
Caffeine	258.094	182.360	2.157	0.001	2.245
Arg-Thr	258.152	351.277	2.282	0.013	1.453
Acadesine (Drug)	259.097	182.402	1.618	0.020	1.589
Tetrahydro-L-biopterin	264.108	160.674	1.882	0.015	1.687

Indolelactic acid	266.099	223.346	1.771	0.018	1.459
Pro-Ser	266.107	317.006	2.273	0.002	1.700
Porphobilinogen	268.123	336.068	1.887	0.028	1.471
Glycitein	285.075	43.463	2.551	0.000	7.006
Pro-Glu	286.139	286.877	1.536	0.036	1.417
Etomidate	286.150	199.050	1.809	0.014	1.606
Pro-Gln	288.089	337.523	1.602	0.022	0.590
Terbutaline	289.154	195.462	1.804	0.014	0.769
Procaterol	291.169	239.327	2.359	0.000	1.521
Diosmetin	301.070	44.517	2.613	0.000	36.418
DL-.alpha.-Phenylglycine	303.133	48.615	2.490	0.000	1.766
Eicosapentaenoic acid	303.230	47.812	1.830	0.027	1.241
Arachidonic Acid (peroxide free)	305.246	45.318	1.623	0.034	1.514
Phenylacetic acid	311.065	37.475	1.653	0.035	1.270
1-Palmitoylglycerol	313.273	181.231	1.651	0.030	2.877
Tyr-Val	325.111	117.567	1.544	0.037	1.448
Eicosapentaenoic Acid ethyl ester	330.263	50.997	1.704	0.050	0.494
Lys-Trp	332.190	365.621	2.141	0.002	1.306
Vincamine	337.197	216.332	2.053	0.028	1.852
Tyr-Asp	338.131	313.025	1.613	0.026	1.508
(-)-Usnic acid	345.095	46.119	1.863	0.025	2.312
Arg-Tyr	360.169	38.334	2.093	0.010	1.454
5-Hydroxydopamine	361.137	51.006	1.981	0.027	4.285
Zotepine	395.093	305.129	1.720	0.046	2.581
Lithocholic acid	399.287	35.143	1.832	0.007	1.459
D-Mannitol	403.113	156.891	1.787	0.016	1.309

RU-0211	408.249	194.079	1.393	0.040	1.683
Dihydrotachysterol	421.344	34.151	1.713	0.040	1.222
6"-O-Acetyldaidzin	423.116	50.422	1.662	0.048	1.438
N-Acetyl-D-lactosamine	428.122	46. 245	1.808	0.034	1.230
Enoxolone	453.333	48.639	1.842	0.029	1.227
Deoxycytidine	455.188	199.028	1.823	0.028	2.560
25-Hydroxycholesterol	463.376	33.061	1.904	0.018	1.197
Diosmin	609.177	190.777	2.562	0.005	38.191

[1]MZ = mass-to-charge ratio.

[2]R.T = represents retention time.

[3]VIP >1 and [4]Pvalue<0.05 are listed in the table. Pvalues were calculated according to Student's T-test (n=6).

[5]FC = fold change. If the fold change value is less than 1, it means that there is less metabolite in the MHB group than in the CON group.

Table S5. Pathway analysis of rumen fluid metabolomics in MHB and CON groups of fattening sheep.

Pathway	Total	Hits[1]	Raw p[2]	Impact[3]	Hits compounds
Pyrimidine metabolism	37	3	0.030	0.010	Deoxycytidine cpd:C00881; Thymidine cpd:C00214; Thymine cpd:C00178
Phenylalanine metabolism	9	1	0.157	0.000	Ortho-Hydroxyphenylacetic acid cpd:C05852
Tryptophan metabolism	41	2	0.177	0.003	L-3-Hydroxykynurenine cpd:C03227; Indoleacetic acid cpd:C00954
Biosynthesis of unsaturated fatty acids	42	2	0.184	0.000	Arachidonic acid cpd:C00219; Eicosapentaenoic acid cpd:C06428
Riboflavin metabolism	11	1	0.188	0.167	Hydroquinone cpd:C00530
Caffeine metabolism	12	1	0.203	0.000	Caffeine cpd:C07481
Porphyrin and chlorophyll metabolism	25	1	0.379	0.042	Porphobilinogen cpd:C00931
Arachidonic acid metabolism	36	1	0.497	0.326	Arachidonic acid cpd:C00219
Valine, leucine and isoleucine degradation	38	1	0.517	0.000	Methylmalonic acid cpd:C02170
Primary bile acid biosynthesis	46	1	0.586	0.011	25-Hydroxycholesterol cpd:C15519
Purine metabolism	68	1	0.732	0.005	Deoxyadenosine cpd:C00559
Taurine and hypotaurine metabolism	7	1	0.078	0.250	3-Sulfinoalanine cpd:C00606
Nicotinate and nicotinamide metabolism	13	1	0.140	0.159	Niacinamide cpd:C00153
Cysteine and methionine metabolism	28	1	0.279	0.023	3-Sulfinoalanine cpd:C00606
Tyrosine metabolism	42	1	0.389	0.000	p-Hydroxyphenylacetic acid cpd:C00642

[1] Hits represent the number of significantly different ruminal metabolites matched in one pathway.

[2] P is the original *p*-Value obtained by pathway analysis.

[3] Impact is the influencing factor of the pathway obtained by topology analysis.

Table S6. Significant differences in metabolites between MHB group and CON group in serum of fattening sheep.

Name	MZ	R.T(min)	VIP	*p*-Value	FC
**Negative**					
Dihydrothymine	187.07	372.27	1.972	0.000	0.276
Allantoate/Allantoic acid	175.05	333.30	2.027	0.000	0.316
Sucrose	342.12	315.47	2.015	0.000	0.215
3,4-Methylenedioxymethamphetamine (MDMA)	214.09	46.74	1.995	0.000	0.362
4-Nitrophenol	138.02	38.01	1.711	0.000	0.500
N4-Acetylcytidine	284.09	158.48	1.968	0.000	3.248
hydrocortisone 21-acetate	425.20	47.98	1.936	0.000	1.583
2-Deoxyribose 5-phosphate	273.04	23.37	1.992	0.001	2.902
N-Formylmethionyl-Leucylphenylalanine	436.20	294.12	1.645	0.001	0.271
MK 571	573.13	333.19	1.907	0.001	0.138
Muramic acid	250.09	352.87	1.987	0.001	0.325
Phosphorylcholine	242.08	373.43	1.750	0.001	0.658
L-homocysteic acid	204.00	32.80	1.873	0.001	1.716
PS(16:0/16:0)	734.52	49.68	1.930	0.001	0.317
all cis-(6,9,12)-Linolenic acid	277.22	44.52	1.816	0.001	1.444
Stavudine	245.06	167.72	1.701	0.002	0.429
Fludrocortisone acetate	403.19	259.11	1.808	0.003	1.765
Arachidic acid	311.29	40.77	1.711	0.003	1.401
Glycerol	92.05	176.40	1.628	0.004	0.581
Glycerol 3-phosphate	171.01	372.99	1.665	0.005	0.628
(R)-mevalonic acid 5-Phosphate	265.09	26.22	2.025	0.005	3.131
D-Sorbitol 6-phosphate	283.13	28.55	1.551	0.005	1.485
D-Ribose 5-phosphate	211.00	24.64	1.740	0.005	0.561

Undecanoic Acid	185.15	46.41	1.586	0.006	0.720
Indoxyl sulfate	212.00	33.38	1.571	0.007	0.679
Arachidonic Acid (peroxide free)	303.23	43.09	1.655	0.007	0.764
Beta-Alanine	177.09	48.39	1.619	0.007	0.512
Formylanthranilic acid	164.03	68.88	1.649	0.008	0.527
5'-O-methylthymidine	256.10	144.42	2.008	0.008	0.106
Aminohippuric acid	193.06	176.38	1.659	0.008	0.611
Glucosamine	238.09	296.55	1.651	0.009	0.615
Sedoheptulose	419.13	207.39	1.593	0.011	2.005
Salsalate	317.06	147.48	1.414	0.011	0.713
Topiramate	338.09	281.59	1.544	0.011	0.480
Sepiapterin	296.10	218.32	1.501	0.014	0.843
Acetylvalerenolic acid	308.18	46.73	1.541	0.015	0.717
Maltitol	344.13	260.68	1.583	0.017	0.630
Cysteinylglycine	194.06	176.35	1.425	0.017	0.633
Hippuric acid	178.05	43.43	1.419	0.019	0.619
Myristic acid	227.20	104.78	1.442	0.019	0.833
alpha-hydroxy myristic acid	243.20	103.29	1.623	0.021	0.537
Lipoamide	221.08	47.50	1.675	0.022	0.221
DL-3-Phenyllactic acid	147.04	106.11	1.408	0.022	0.549
2E-Eicosenoic acid	309.28	41.85	1.429	0.023	1.269
1-Palmitoyl-2-hydroxy-sn-glycero-3-phosphoethanolamine	452.28	185.40	1.469	0.026	1.420
N-Acetylneuraminic acid	290.09	325.58	1.444	0.028	0.850
Kynurenic acid	188.03	174.18	1.418	0.032	0.778
2'-O-methylinosine	281.09	143.85	1.398	0.037	1.234
Confertifoline	233.15	30.74	1.096	0.039	2.198

N-Acetyl-L-aspartic acid	174.04	361.34	1.546	0.040	0.617
Indoleacrylic acid	186.06	29.33	1.746	0.042	3.224
N-Acetyl-L-tyrosine	222.08	204.06	1.369	0.044	0.649
Citramalic acid	207.05	287.75	1.419	0.047	1.637
**Postive**					
Val-Gln	246.14	313.23	2.048	0.000	0.114
DL-a-Hydroxybutyric acid	149.02	33.17	2.111	0.000	0.452
Phe-Tyr	328.14	280.88	2.024	0.000	0.226
(-)-Medicarpin	331.11	372.18	1.982	0.000	0.285
Pyridostigmine cation	204.09	252.60	2.016	0.000	0.345
Phe-Cys	310.13	281.00	2.002	0.000	0.326
3,4-Dihydroxyphenylacetic acid	186.07	252.61	2.032	0.000	0.374
Tyr-Phe	311.13	370.99	2.063	0.000	0.402
beta-Hydroxybutyrate	168.06	252.50	2.005	0.000	0.306
Larixinic Acid	144.06	252.80	1.970	0.000	0.257
Phthalic acid Mono-2-ethylhexyl Ester	279.16	33.25	2.130	0.000	0.438
1-Stearoyl-2-arachidonoyl-sn-glycerol	627.53	46.64	2.183	0.000	0.090
Phenoxybenzamine	367.15	282.73	1.948	0.000	0.240
Ile-Thr	296.16	294.16	1.955	0.000	0.385
Pro-Met	310.11	409.32	1.984	0.000	0.343
Val-Tyr	280.14	313.22	1.938	0.000	0.276
Ile-Ser	282.14	313.20	1.947	0.000	0.446
Visnadin	353.13	293.86	2.008	0.000	0.282
Arg-Cys	338.15	393.08	1.982	0.000	0.326
Famciclovir	344.13	315.33	1.960	0.000	0.217
1-Aminocyclopropanecarboxylic acid	162.08	252.80	1.929	0.000	0.374

Enoxacin	338.16	353.82	1.960	0.000	0.361
His-Tyr	318.13	410.89	1.896	0.000	0.486
Arg-Tyr	337.17	450.65	1.932	0.000	0.361
Kynuramine	187.09	283.93	2.144	0.000	0.127
Tyr-Lys	309.16	439.95	1.940	0.000	0.398
beta-Octylglucoside	337.16	354.30	1.954	0.000	0.315
D-gluconate	238.09	368.00	1.906	0.000	0.296
Arg-Thr	339.17	451.27	1.931	0.000	0.429
1,7-Dimethyluric acid	260.07	370.20	1.864	0.000	0.369
Hippuric acid	180.06	174.76	1.983	0.000	1.471
Acetylglycine	118.05	368.04	1.826	0.000	0.348
Flumequine	261.08	370.43	1.891	0.000	0.482
Caproic acid	158.12	47.43	1.961	0.000	2.066
Met-Tyr	312.11	306.24	1.874	0.000	0.310
2-Methylguanosine	298.11	218.30	1.875	0.000	0.824
Argininosuccinic acid	313.11	306.24	1.790	0.000	0.348
Thr-Val	218.12	390.98	1.910	0.000	0.437
DL-Vanillylmandelic acid	216.09	352.43	1.972	0.001	0.400
Gly-Glu	246.11	365.74	1.578	0.001	0.491
Glycine	76.04	368.02	1.795	0.001	0.463
D-Ribulose 1,5-bisphosphate	328.02	352.74	1.921	0.001	0.472
Diethanolamine	88.08	211.63	2.151	0.001	21.539
Dihydro-4,4-dimethyl-2,3-furandione	146.08	321.22	1.816	0.001	0.815
N4-Acetylcytidine	286.10	158.14	1.811	0.001	2.453
4-Oxoretinol	301.21	35.35	1.878	0.002	1.806
Pyridoxine	211.11	85.62	1.702	0.002	0.563

1-Oleoyl-L-.alpha.-lysophosphatidic acid	478.29	184.67	1.774	0.002	1.547
3-Aminobenzoic acid	120.04	176.34	1.571	0.002	0.558
Acetyl-DL-Valine	160.10	313.75	1.381	0.002	0.497
Tyr-Glu	371.14	294.15	2.022	0.003	0.219
Gamma-Glutamylcysteine	292.10	409.54	1.766	0.003	0.581
.beta.-Cyano-L-alanine	175.07	209.01	1.744	0.003	0.668
Acetylcarnitine	204.12	293.42	1.805	0.003	1.823
Cortexolone	385.17	364.67	1.837	0.003	0.377
Zolmitriptan	326.13	434.96	1.768	0.003	0.507
Glycerophosphocholine	258.11	372.06	1.821	0.003	0.528
Kynurenic acid	190.05	173.80	1.670	0.003	0.623
5-Methylcytidine	258.11	194.03	1.597	0.003	0.766
Acadesine (Drug)	259.10	190.63	1.680	0.004	0.635
Valproic acid	186.15	37.65	1.879	0.004	2.070
1-Palmitoyl-2-hydroxy-sn-glycero-3-phosphoethanolamine	454.29	185.84	1.709	0.005	1.522
Gly-Arg	295.15	433.33	1.693	0.005	0.447
L-Pipecolic acid	171.11	252.57	1.960	0.006	0.179
Pyruvaldehyde	145.05	293.42	1.831	0.006	1.597
2-Ethoxyethanol	151.10	63.93	1.573	0.006	0.604
Met-Met	344.10	323.63	1.705	0.007	0.512
His-Ser	225.10	303.66	1.907	0.008	2.597
(+-)5,6-DHET	356.28	83.48	1.930	0.008	0.150
Pelletierine	205.13	293.42	1.803	0.009	1.866
Omeprazole	310.09	364.90	1.659	0.010	0.482
Pargyline	336.25	44.39	1.582	0.010	1.338
Oxindole	194.08	204.58	1.534	0.012	0.765

Ile-Asn	284.10	371.61	1.648	0.014	0.615
Crotonic acid	128.07	251.66	1.492	0.014	0.732
alpha-N-Acetyl-L-glutamine	211.07	288.73	1.514	0.014	0.715
D-Glucono-1,5-lactone	220.08	394.62	1.396	0.015	0.564
2-Methyl-3-hydroxybutyric acid	259.12	28.24	1.416	0.015	1.599
Troglitazone	459.21	338.33	1.886	0.015	0.138
N-Acetylmannosamine	260.05	29.54	1.391	0.015	0.492
Levonordefrin	148.07	171.38	1.418	0.016	0.766
20-Hydroxyarachidonic acid	362.27	43.41	1.796	0.018	0.539
Altretamine	228.20	37.41	1.417	0.018	1.777
Ile-Tyr	294.15	340.46	1.518	0.019	0.501
N-.alpha.-Acetyl-L-arginine	217.13	355.67	1.567	0.020	0.597
His-Thr	257.12	346.14	1.519	0.021	0.615
L-Carnitine	162.11	292.16	1.418	0.024	0.810
Quinate	234.10	394.49	1.542	0.025	0.693
Itaconic acid	172.06	288.73	1.391	0.025	0.698
PC(16:0/16:0)	756.55	137.01	1.509	0.025	1.248
Urea	61.04	105.91	1.415	0.026	0.907
Taurochenodeoxycholate	482.29	142.18	1.430	0.026	0.593
3-Ureidopropionate	133.06	295.32	1.365	0.027	0.812
Desmosterol	385.34	34.02	1.237	0.030	1.234
cis-4-Hydroxy-D-proline	173.09	340.80	1.484	0.030	0.601
Behenic acid	358.37	51.57	1.208	0.032	0.562
N-Oleoylethanolamine	326.30	35.30	1.494	0.035	1.358
Lys-Cys	291.15	464.66	1.390	0.036	0.661
Jasmine lactone	354.26	44.13	1.387	0.036	1.225

Decanoyl-L-carnitine	316.25	177.39	1.200	0.036	0.780
Taurodeoxycholic acid	500.30	142.50	1.388	0.038	0.574
Betaine aldehyde	102.09	343.59	1.247	0.040	0.869
Erythritol	186.07	147.14	1.198	0.041	0.879
Methoxyacetic acid	181.07	174.53	1.504	0.042	1.550
Metronidazole	172.07	37.71	1.480	0.042	0.328
2E-Eicosenoic acid	377.23	34.72	1.608	0.044	0.247
Imidazole	110.07	25.89	1.320	0.045	0.753
Scopoline	156.10	351.74	1.312	0.046	0.766
Diethylcarbamazine	241.20	27.69	1.219	0.048	0.845
PC(20:5(5Z,8Z,11Z,14Z,17Z)/20:5(5Z,8Z,11Z,14Z,17Z))	809.54	46.50	1.423	0.050	67.81

Table S7. Pathway analysis of serum metabolomics in MHB and CON groups of fattening sheep.

Pathway	Total	Hits	Raw p	Impact	Hits compunds
Glycine, serine and threonine metabolism	32	3	0.038	0.292	Glycine cpd:C00037; Betaine aldehyde cpd:C00576; Pyruvaldehyde cpd:C00546
Cyanoamino acid metabolism	6	1	0.134	0.000	Glycine cpd:C00037
Methane metabolism	9	1	0.195	0.000	Glycine cpd:C00037
Nitrogen metabolism	9	1	0.195	0.000	Glycine cpd:C00037
Vitamin B6 metabolism	9	1	0.194	0.078	Pyridoxine cpd:C00314
Arginine and proline metabolism	44	2	0.281	0.022	Argininosuccinic acid cpd:C03406; Urea cpd:C00086
Primary bile acid biosynthesis	46	2	0.299	0.060	Glycine cpd:C00037; Taurochenodesoxycholic acid cpd:C05465
Pantothenate and CoA biosynthesis	15	1	0.304	0.041	Ureidopropionic acid cpd:C02642
beta-Alanine metabolism	17	1	0.337	0.222	Ureidopropionic acid cpd:C02642
Pentose phosphate pathway	19	1	0.368	0.000	Gluconolactone cpd:C00198
Propanoate metabolism	20	1	0.383	0.000	2-Hydroxybutyric acid cpd:C05984
Pyruvate metabolism	22	1	0.413	0.054	Pyruvaldehyde cpd:C00546
Alanine, aspartate and glutamate metabolism	23	1	0.427	0.020	Argininosuccinic acid cpd:C03406
Porphyrin and chlorophyll metabolism	25	1	0.454	0.000	Glycine cpd:C00037
Glutathione metabolism	26	1	0.467	0.006	Glycine cpd:C00037

Glycerophospholipid metabolism	29	1	0.505	0.024	Glycerophosphocholine cpd:C00670
Steroid biosynthesis	35	1	0.573	0.000	Desmosterol cpd:C01802
Pyrimidine metabolism	37	1	0.594	0.021	Ureidopropionic acid cpd:C02642
Amino sugar and nucleotide sugar metabolism	37	1	0.594	0.142	N-Acetylmannosamine cpd:C00645
Biosynthesis of unsaturated fatty acids	42	1	0.641	0.000	Behenic acid cpd:C08281
Tyrosine metabolism	42	1	0.641	0.001	3,4-Dihydroxybenzeneacetic acid cpd:C01161
Drug metabolism - cytochrome P450	56	1	0.746	0.000	Valproic acid cpd:C07185
Aminoacyl-tRNA biosynthesis	64	1	0.793	0.000	Glycine cpd:C00037
Purine metabolism	68	1	0.812	0	Urea cpd:C00086
Glycerolipid metabolism	18	2	0.040	0.307	Glycerol 3-phosphate cpd:C00093; Glycerol cpd:C00116
Terpenoid backbone biosynthesis	15	1	0.239	0.172	Mevalonic acid-5P cpd:C01107
Galactose metabolism	26	1	0.379	0.000	Glycerol cpd:C00116
Arachidonic acid metabolism	36	1	0.484	0.326	Arachidonic acid cpd:C00219
Fatty acid biosynthesis	38	1	0.503	0.000	Myristic acid cpd:C06424
Tryptophan metabolism	41	1	0.530	0.010	Formylanthranilic acid cpd:C05653

Table S8. Significant differences in metabolites between MHB and CON groups in urine of fattening sheep.

Name	MZ	R.T(min)	VIP	*p*-Value	FC
**Negative**					
Lecanoric acid	377.10	286.52	2.345	0.000	3.326
1-Naphthol	160.08	172.93	2.340	0.000	2.066
Ramipril	415.23	215.71	2.535	0.001	27.859
Aminohippuric acid	193.06	180.96	2.146	0.002	0.364
Cysteinylglycine	194.06	180.90	2.135	0.003	0.373
Ribothymidine	274.11	45.75	2.423	0.003	2.808
17.beta.-Estradiol 3-.beta.-D-glucuronide	507.22	234.90	2.369	0.003	5.613
DL-O-tyrosine	361.15	288.72	2.412	0.004	12.262
Clozapine	325.13	46.37	1.805	0.006	0.556
3-Phosphoserine	185.01	71.82	1.936	0.010	0.474
N-Acetyl-L-phenylalanine	206.08	172.31	1.876	0.013	1.456
Succinylacetone	217.07	133.12	1.944	0.013	1.968
Kainic Acid	234.08	69.44	1.968	0.014	3.010
Pectin (Galacturonic acid)	193.03	216.96	1.973	0.019	3.663
4-Hydroxybenzaldehyde	243.07	46.27	1.660	0.021	0.604
Dicumarol	335.05	102.25	1.649	0.023	0.646
Estrone-3-glucuronide	445.18	215.71	1.891	0.024	11.010
5'-Deoxyadenosine	250.09	208.73	1.899	0.025	1.598
Phosphocreatine	211.04	277.95	1.665	0.032	0.469
gamma-Glutamyl-L-methionine	259.07	269.99	1.828	0.036	0.535
Traumatic Acid	265.09	28.58	2.253	0.040	5.034
3-Isopropylmalate	157.05	131.27	1.478	0.042	0.615
O-Phospho-L-threonine	199.03	51.15	1.979	0.042	0.508

Acetohexamide	361.06	209.47	1.264	0.047	7.028
**Postive**					
4-Hydroxy-3-methoxycinnamaldehyde	217.03	202.03	2.851	0.000	6.249
Imidazole	132.05	172.71	2.315	0.000	2.510
gamma-Aminobutryic acid	121.10	34.18	2.451	0.001	1.793
Jasmine lactone	151.11	49.32	2.699	0.002	3.235
Allocystathionine	187.05	271.16	2.170	0.002	2.925
alpha-Farnesene	205.19	214.87	2.199	0.002	2.188
Oxaloacetate	193.03	215.36	2.697	0.003	8.740
Benzylazanium	149.11	28.68	2.732	0.003	4.507
Lavandulol	137.13	222.76	2.762	0.004	8.251
Hippuric acid	180.07	179.65	1.784	0.006	2.338
5'-Deoxyadenosine	269.13	234.70	2.415	0.006	0.680
4-Hydroxybutanoic acid lactone	128.07	78.51	2.231	0.009	1.982
Ser-Lys	310.05	338.55	2.259	0.014	0.371
2'-O-methylcytidine	258.11	168.18	1.841	0.015	1.484
(S)-(-)-Citronellic acid	171.14	217.37	2.664	0.016	5.566
3-Mercapto-2-butanone	247.03	90.21	2.188	0.017	0.508
Pro-Ala	204.13	322.10	2.018	0.020	1.482
Midazolam	389.09	43.45	1.988	0.020	0.537
Tyr-Gln	292.13	33.61	1.934	0.021	0.602
Sumatriptan	340.11	399.92	2.038	0.022	1.640
Anthranilic acid (Vitamin L1)	138.05	274.40	2.560	0.023	0.374
Gly-Glu	265.11	228.71	2.108	0.030	0.713
2'-Deoxy-D-ribose	134.06	179.86	1.595	0.031	1.430
1-Aminocyclopropanecarboxylic acid	143.08	321.82	2.402	0.032	0.585

Mirtazapine	266.16	27.97	2.257	0.035	2.231
Mevalonic acid	319.14	110.84	1.966	0.036	5.642
L-Valine	235.17	49.40	1.548	0.038	0.570
Xylitol	153.07	283.42	1.502	0.039	1.482
Larixinic Acid	127.04	37.49	1.787	0.040	2.168
Securinine	240.10	240.73	1.309	0.048	1.541

Table S9. Pathway analysis of urine metabolomics in MHB and CON groups of fattening sheep.

Pathway	Total	Hits	Raw p	Impact	Hits compunds
Valine, leucine and isoleucine biosynthesis	11	1	0.069	0.333	L-Valine cpd:C00183
Pentose and glucuronate interconversions	15	1	0.093	0.000	D-Xylitol cpd:C00379
Pantothenate and CoA biosynthesis	15	1	0.093	0.000	L-Valine cpd:C00183
Terpenoid backbone biosynthesis	15	1	0.093	0.145	Mevalonic acid cpd:C00418
Glyoxylate and dicarboxylate metabolism	16	1	0.099	0.148	Oxalacetic acid cpd:C00036
Pentose phosphate pathway	19	1	0.117	0.000	Deoxyribose cpd:C01801
Citrate cycle (TCA cycle)	20	1	0.123	0.114	Oxalacetic acid cpd:C00036
Pyruvate metabolism	22	1	0.134	0.015	Oxalacetic acid cpd:C00036
Alanine, aspartate and glutamate metabolism	23	1	0.140	0.087	Oxalacetic acid cpd:C00036
Glycolysis or Gluconeogenesis	26	1	0.157	0.000	Oxalacetic acid cpd:C00036
Valine, leucine and isoleucine degradation	38	1	0.221	0.000	L-Valine cpd:C00183
Aminoacyl-tRNA biosynthesis	64	1	0.346	0.000	L-Valine cpd:C00183
Glutathione metabolism	26	1	0.157	0.008	Cysteinylglycine cpd:C01419
Arginine and proline metabolism	44	1	0.251	0	Phosphocreatine cpd:C02305
Steroid hormone biosynthesis	67	1	0.360	0	Estrone glucuronide cpd:C11133

Table S10. Analysis of significant differential expression of circRNA between MHB group and CON group.

circRNA id[1]	MeanTPM[2] (MHB)	MeanTPM (CON)	log2FoldChange[3]	*p*-Value[4]
**UP**				
circRNA05275	53.586	0.000	19.032	0.032
circRNA01712	930.87	175.609	2.406	0.042
circRNA00452	258.294	100.547	1.361	0.045
circRNA01373	473.793	0.000	22.176	0.0148
circRNA00282	515.822	0.000	22.298	0.011
circRNA01037	1960.256	0.000	24.225	0.045
circRNA04879	305.979	0.000	21.545	0.025
circRNA04936	61.442	0.000	19.229	0.017
circRNA01358	823.354	0.000	22.973	0.036
**Down**				
circRNA01240	273.720	861.046	-1.653	0.044
circRNA04405	0.000	69.112	-19.399	0.0165
circRNA04145	0.000	649.119	-22.630	0.0379
circRNA01788	289.793	2644.997	-3.190	0.0229
circRNA03991	0.000	137.537	-20.391	0.041

[1] circRNA ID: Transcript number.

[2] MeanTPM: Expression level of grouping.

[3] log2FoldChange: log2 value of difference multiple.

[4] *p*-Value: Statistical significance test indicators.

Table S11. 30 significantly enriched differential circRNAs in the MHB diet group.

GO.ID[1]	Term[2]	Ontology[3]	Significant[4]	Annotated[5]	*p*-Value
GO:0070498	interleukin-1-mediated signaling pathway	biological process	2/11	17/14620	0.000
GO:0071347	cellular response to interleukin-1	biological process	2/11	46/14620	0.001
GO:0043990	histone H2A-S1 phosphorylation	biological process	1/11	1/14620	0.001
GO:0070555	response to interleukin-1	biological process	2/11	56/14620	0.001
GO:0019276	UDP-N-acetylgalactosamine metabolic process	biological process	1/11	3/14620	0.002
GO:0043987	histone H3-S10 phosphorylation	biological process	1/11	3/14620	0.002
GO:0043988	histone H3-S28 phosphorylation	biological process	1/11	3/14620	0.002
GO:1990164	histone H2A phosphorylation	biological process	1/11	3/14620	0.002
GO:0006047	UDP-N-acetylglucosamine metabolic process	biological process	1/11	4/14620	0.003
GO:1902083	negative regulation of peptidyl-cysteine S-nitrosylation	biological process	1/11	4/14620	0.003
GO:0033129	positive regulation of histone phosphorylation	biological process	1/11	5/14620	0.004
GO:0071447	cellular response to hydroperoxide	biological process	1/11	5/14620	0.004
GO:2000169	regulation of peptidyl-cysteine S-nitrosylation	biological process	1/11	5/14620	0.004
GO:2000659	regulation of interleukin-1-mediated signaling pathway	biological process	1/11	5/14620	0.004
GO:0001842	neural fold formation	biological process	1/11	6/14620	0.005
GO:1905064	negative regulation of vascular smooth muscle cell differentiation	biological process	1/11	6/14620	0.005
GO:0035404	histone-serine phosphorylation	biological process	1/11	7/14620	0.005
GO:0030259	lipid glycosylation	biological process	1/11	8/14620	0.006
GO:0034599	cellular response to oxidative stress	biological process	2/11	168/14620	0.007
GO:0017014	protein nitrosylation	biological process	1/11	9/14620	0.007

GO:0018119	peptidyl-cysteine S-nitrosylation	biological process	1/11	9/14620	0.007
GO:1905063	regulation of vascular smooth muscle cell differentiation	biological process	1/11	9/14620	0.007
GO:0033127	regulation of histone phosphorylation	biological process	1/11	10/14620	0.008
GO:0033194	response to hydroperoxide	biological process	1/11	11/14620	0.008
GO:0051151	negative regulation of smooth muscle cell differentiation	biological process	1/11	12/14620	0.009
GO:1901741	positive regulation of myoblast fusion	biological process	1/11	12/14620	0.009
GO:1901739	regulation of myoblast fusion	biological process	1/11	15/14620	0.011
GO:1903204	negative regulation of oxidative stress-induced neuron death	biological process	1/11	16/14620	0.012
GO:0035886	vascular smooth muscle cell differentiation	biological process	1/11	18/14620	0.013
GO:0060143	positive regulation of syncytium formation by plasma membrane fusion	biological process	1/11	19/14620	0.014

[1] GO.ID：Number and name of GO.

[2] Term：Description information of GO function.

[3] Ontology：Category of GO（cellular component；biological process；molecular function）.

[4] Significant：Number of differentially expressed circRNA host genes annotated to the GO/Number of differentially expressed circRNA host genes annotated to the GO database.

[5] Annotated：Number of genes annotated to the GO/Number of genes annotated to the GO database.

Table S12. Significant differential expression analysis of transcripts (lncRNA, mRNA) between MHB group and CON group.

**Transcript id**[1]	**MeanTPM (MHB)**	**MeanTPM (CON)**	**log2FoldChange**	***p*-Value**
**UP**				
MSTRG.86362.1	30.193	0.000	18.204	0.000
ENSOART00000000780	25.157	3.633	2.792	0.000
ENSOART00000017401	10.127	3.757	1.431	0.000
ENSOART00000008854	14.040	1.057	3.732	0.000
ENSOART00000020109	20.330	3.070	2.727	0.000
ENSOART00000014168	8.613	2.243	1.941	0.000
ENSOART00000014772	7.207	0.987	2.869	0.000
ENSOART00000016187	7.333	1.877	1.966	0.000
ENSOART00000005238	5.547	0.320	4.115	0.000
ENSOART00000016232	17.523	0.057	8.273	0.000
**Down**				
MSTRG.26668.6	0.000	32.857	-18.326	0.000
MSTRG.87688.2	0.080	14.313	-7.483	0.000
MSTRG.90841.1	0.570	6.277	-3.461	0.000
ENSOART00000008940	2.140	9.407	-2.136	0.000
ENSOART00000017457	0.000	10.403	-16.667	0.000
ENSOART00000000735	0.000	7.543	-16.203	0.000
ENSOART00000006877	0.923	14.480	-3.971	0.000
ENSOART00000006875	0.001	10.270	-16.648	0.000
ENSOART00000018756	0.657	52.057	-6.309	0.000
ENSOART00000011885	0.000	5.317	-15.698	0.000
ENSOART00000017226	1.967	6.463	-1.717	0.000
ENSOART00000003468	0.000	64.963	-19.309	0.000
ENSOART00000022712	202.550	422.400	-1.060	0.000
ENSOART00000005620	56.587	277.410	-2.293	0.000
ENSOART00000007695	12.797	33.660	-1.395	0.000
ENSOART00000014234	0.030	37.867	-10.302	0.000
ENSOART00000015276	42.830	98.020	-1.194	0.000

[1]Transcript ID：Transcript number.

Table S13. Gene functions corresponding to 30 significantly enriched differential transcripts in the MHB group.

GO.ID	Term	Qntology	Significant	Annotated	*p*-Value
GO:0090049	regulation of cell migration involved in sprouting angiogenesis	biological process	2/19	30/14620	0.001
GO:0002042	cell migration involved in sprouting angiogenesis	biological process	2/19	41/14620	0.001
GO:0034395	regulation of transcription from RNA polymerase II promoter in response to iron	biological process	1/19	1/14620	0.001
GO:0036335	intestinal stem cell homeostasis	biological process	1/19	1/14620	0.001
GO:0030336	negative regulation of cell migration	biological process	3/19	179/14620	0.002
GO:1903670	regulation of sprouting angiogenesis	biological process	2/19	46/14620	0.002
GO:2000146	negative regulation of cell motility	biological process	3/19	189/14620	0.002
GO:0010631	epithelial cell migration	biological process	3/19	201/14620	0.002
GO:0051271	negative regulation of cellular component movement	biological process	3/19	202/14620	0.002
GO:0090132	epithelium migration	biological process	3/19	203/14620	0.002
GO:0009408	response to heat	biological process	2/19	54/14620	0.002
GO:0090130	tissue migration	biological process	3/19	208/14620	0.002
GO:0040013	negative regulation of locomotion	biological process	3/19	211/14620	0.002
GO:0006788	heme oxidation	biological process	1/19	2/14620	0.003
GO:0060995	cell-cell signaling involved in kidney development	biological process	1/19	2/14620	0.003
GO:0061289	Wnt signaling pathway involved in kidney development	biological process	1/19	2/14620	0.003
GO:0061290	canonical Wnt signaling pathway involved in	biological process	1/19	2/14620	0.003

	metanephric kidney development				
GO:0072204	cell-cell signaling involved in metanephros development	biological process	1/19	2/14620	0.003
GO:0106004	tRNA (guanine-N7)-methylation	biological process	1/19	2/14620	0.003
GO:0043535	regulation of blood vessel endothelial cell migration	biological process	2/19	68/14620	0.003
GO:0019276	UDP-N-acetylgalactosamine metabolic process	biological process	1/19	3/14620	0.004
GO:0072718	response to cisplatin	biological process	1/19	3/14620	0.004
GO:0072719	cellular response to cisplatin	biological process	1/19	3/14620	0.004
GO:2001013	epithelial cell proliferation involved in renal tubule morphogenesis	biological process	1/19	3/14620	0.004
GO:0002040	sprouting angiogenesis	biological process	2/19	74/14620	0.004
GO:0006979	response to oxidative stress	biological process	3/19	256/14620	0.004
GO:0001667	ameboidal-type cell migration	biological process	3/19	271/14620	0.005
GO:0042303	molting cycle	biological process	2/19	82/14620	0.005
GO:0042633	hair cycle	biological process	2/19	82/14620	0.005
GO:0006047	UDP-N-acetylglucosamine metabolic process	biological process	1/19	4/14620	0.005

Table S14. Analysis of significant differences in miRNA expression between MHB group and CON group.

Id[1]	Log2FoldChange[2]	Log2CPM[3]	*p*-Value
**Up**			
aae-miR-124	4.574	-1.234	0.001
aca-miR-124a	4.574	-1.234	0.001
aca-miR-124b	4.934	-1.293	0.002
aca-miR-191-3p	1.536	-2.902	0.027
aca-miR-34a-5p	3.375	-1.478	0.019
aca-miR-34c-5p	3.375	-1.478	0.019
aga-miR-124	4.574	-1.234	0.001
age-miR-124a	4.844	-0.794	0.001
aja-miR-21	1.429	1.818	0.010
ame-miR-124	4.574	-1.234	0.001
api-miR-124	4.574	-1.234	0.001
asu-miR-124-3p	4.574	-1.234	0.001
bbe-miR-124-3p	4.574	-1.234	0.001
bfl-miR-124-3p	4.574	-1.234	0.001
bma-miR-124	4.574	-1.234	0.001
bmo-miR-124	4.574	-1.234	0.001
bmo-miR-2779	1.933	6.141	0.017
bta-miR-124a	4.574	-1.234	0.001
bta-miR-124b	4.574	-1.234	0.001
bta-miR-1814c	3.608	-4.041	0.010
bta-miR-21-3p	1.323	2.304	0.006
bta-miR-2310	3.608	-4.041	0.010
bta-miR-2318	4.338	-3.767	0.023
bta-miR-2404	2.367	-2.346	0.017
bta-miR-2478	1.087	7.090	0.011
bta-miR-34c	3.375	-1.478	0.019
bta-miR-412	1.724	-3.596	0.050
cbn-miR-124	4.574	-1.234	0.001
cbr-miR-124a	4.574	-1.234	0.001
cel-miR-124-3p	4.574	-1.234	0.001
cfa-miR-124	4.574	-1.234	0.001
cfa-miR-146a	2.455	2.187	0.008
cfa-miR-34c	3.493	1.219	0.023
cgr-miR-124	4.574	-1.234	0.001
cgr-miR-146a	2.455	2.187	0.008
cgr-miR-21-3p	1.429	1.818	0.010
cgr-miR-322-5p	2.174	-2.821	0.045
cgr-miR-34c-5p	3.493	1.219	0.023
cgr-miR-412-3p	1.724	-3.596	0.050
cgr-miR-744-3p	1.659	-2.912	0.048

chi-miR-124a	4.574	-1.234	0.001
chi-miR-1814	3.608	-4.041	0.010
chi-miR-21-3p	1.429	1.818	0.010
chi-miR-2318	4.338	-3.767	0.023
chi-miR-2404	2.367	-2.346	0.017
chi-miR-34c-5p	3.493	1.219	0.023
chi-miR-412-3p	1.724	-3.596	0.050
cin-miR-124-3p	4.574	-1.234	0.001
cqu-miR-124	4.574	-1.234	0.001
crm-miR-124a	4.574	-1.234	0.001
csa-miR-124	4.574	-1.234	0.001
cte-miR-124	4.574	-1.234	0.001
dan-miR-124	4.574	-1.234	0.001
der-miR-124	4.574	-1.234	0.001
dgr-miR-124	4.574	-1.234	0.001
dme-miR-124-3p	4.574	-1.234	0.001
dmo-miR-124	4.574	-1.234	0.001
dpe-miR-124	4.574	-1.234	0.001
dps-miR-124	4.574	-1.234	0.001
dpu-miR-124	4.574	-1.234	0.001
dre-miR-124-3p	4.574	-1.234	0.001
dse-miR-124	4.574	-1.234	0.001
dsi-miR-124	4.574	-1.234	0.001
dvi-miR-124-3p	4.574	-1.234	0.001
dwi-miR-124	4.574	-1.234	0.001
dya-miR-124	4.574	-1.234	0.001
eca-miR-124	4.574	-1.234	0.001
eca-miR-146a	2.455	2.187	0.008
eca-miR-34c	3.493	1.219	0.023
efu-miR-124	4.844	-0.794	0.001
efu-miR-21	1.429	1.818	0.010
efu-miR-9277	1.473	1.818	0.029
egr-miR-124a	4.574	-1.234	0.001
emu-miR-124a	4.574	-1.234	0.001
fru-miR-124	4.574	-1.234	0.001
gga-miR-124a-3p	4.844	-0.794	0.001
gga-miR-146a-5p	2.455	2.187	0.008
gga-miR-34b-5p	3.375	-1.478	0.019
gga-miR-34c-5p	3.493	1.219	0.023
ggo-miR-124a	4.844	-0.794	0.001
ggo-miR-146a	2.455	2.187	0.008
ggo-miR-34c	3.375	-1.478	0.019
ggo-miR-503	4.767	-4.230	0.049
hco-miR-124	4.574	-1.234	0.001

hme-miR-124	4.574	-1.234	0.001
hsa-miR-124-3p	4.574	-1.234	0.001
hsa-miR-146a-5p	2.455	2.187	0.008
hsa-miR-21-3p	4.094	-2.872	0.002
hsa-miR-34c-5p	3.493	1.219	0.023
hsa-miR-4454	1.002	9.396	0.043
hsa-miR-4508	3.799	-3.380	0.001
hsa-miR-503-5p	4.767	-4.230	0.049
hsa-miR-744-3p	1.659	-2.912	0.048
hsa-miR-7975	1.133	3.393	0.024
hsa-miR-7977	1.226	0.112	0.017
ipu-miR-124a	4.574	-1.234	0.001
ipu-miR-7550	1.933	6.140	0.017
isc-miR-124	4.574	-1.234	0.001
lgi-miR-124	4.574	-1.234	0.001
lla-miR-124a	4.844	-0.794	0.001
lva-miR-124-3p	4.574	-1.234	0.001
mdo-miR-124a-3p	4.844	-0.794	0.001
mdo-miR-146a-5p	2.455	2.187	0.008
mdo-miR-34b-5p	3.387	-1.380	0.025
mdo-miR-34c-5p	3.493	1.219	0.023
Metazoa-novel-1-mature	2.447	6.362	0.000
Metazoa-novel-104-mature	1.656	-3.113	0.041
Metazoa-novel-107-mature	4.894	1.005	0.000
Metazoa-novel-108-mature	2.145	-1.992	0.009
Metazoa-novel-113-mature	1.952	-1.624	0.002
Metazoa-novel-114-mature	4.894	1.005	0.000
Metazoa-novel-125-mature	1.484	-2.277	0.018
Metazoa-novel-129-star	4.749	-4.240	0.030
Metazoa-novel-150-mature	3.489	-4.060	0.007
Metazoa-novel-155-mature	1.324	4.098	0.010
Metazoa-novel-175-mature	1.862	6.518	0.001
Metazoa-novel-214-	1.106	7.065	0.010

mature			
Metazoa-novel-234-mature	1.761	0.556	0.013
Metazoa-novel-251-mature	2.040	-1.905	0.038
Metazoa-novel-254-mature	1.450	-2.883	0.046
Metazoa-novel-258-mature	4.318	-1.330	0.008
Metazoa-novel-276-mature	2.107	-0.097	0.024
Metazoa-novel-285-mature	2.780	-3.979	0.044
Metazoa-novel-30-mature	3.238	-3.833	0.024
Metazoa-novel-30-star	3.544	-3.607	0.014
Metazoa-novel-300-mature	1.929	1.898	0.000
Metazoa-novel-307-mature	1.144	8.190	0.002
Metazoa-novel-310-mature	2.471	-1.075	0.013
Metazoa-novel-313-mature	1.457	-0.213	0.037
Metazoa-novel-318-mature	2.690	-0.404	0.000
Metazoa-novel-320-mature	1.143	8.199	0.002
Metazoa-novel-322-mature	3.501	-2.703	0.000
Metazoa-novel-327-mature	1.254	8.903	0.001
Metazoa-novel-331-mature	1.144	8.191	0.002
Metazoa-novel-343-mature	2.726	-4.341	0.042
Metazoa-novel-345-mature	1.550	2.167	0.040
Metazoa-novel-354-mature	1.116	4.763	0.010
Metazoa-novel-359-mature	2.726	-4.341	0.042
Metazoa-novel-362-mature	1.148	3.420	0.022

Metazoa-novel-391-mature	1.111	5.244	0.006
Metazoa-novel-411-mature	1.680	0.821	0.018
Metazoa-novel-417-mature	1.481	3.766	0.005
Metazoa-novel-419-mature	1.144	8.193	0.002
Metazoa-novel-58-mature	5.510	-3.885	0.001
Metazoa-novel-7-mature	1.486	1.275	0.012
Metazoa-novel-72-mature	4.662	-4.259	0.034
Metazoa-novel-8-mature	1.569	1.615	0.005
Metazoa-novel-99-mature	4.163	-3.630	0.003
mml-miR-124a-3p	4.844	-0.794	0.001
mml-miR-146a-5p	2.455	2.187	0.008
mml-miR-21-3p	4.094	-2.872	0.002
mml-miR-34c-5p	3.493	1.219	0.023
mml-miR-503-5p	4.767	-4.230	0.049
mmu-miR-124-3p	4.574	-1.234	0.001
mmu-miR-146a-5p	2.455	2.187	0.008
mmu-miR-21a-3p	1.429	1.818	0.010
mmu-miR-322-5p	2.174	-2.821	0.045
mmu-miR-34c-5p	3.493	1.219	0.023
mmu-miR-3963	1.087	7.090	0.011
mmu-miR-3968	1.101	4.872	0.012
mmu-miR-5119	3.167	-4.181	0.014
mmu-miR-744-3p	1.659	-2.912	0.048
mse-miR-124	4.574	-1.234	0.001
mse-miR-2779	1.933	6.141	0.017
nvi-miR-124	4.574	-1.234	0.001
oan-miR-124a-1-3p	4.574	-1.234	0.001
oan-miR-133c	4.597	-4.307	0.014
oan-miR-1386	1.303	6.736	0.018
oan-miR-21-3p	1.429	1.818	0.010
oan-miR-34a-5p	3.493	1.219	0.023
oan-miR-34b-5p	3.215	-1.614	0.025
odi-miR-124a	4.934	-1.293	0.002
oha-miR-124	4.574	-1.234	0.001
oha-miR-124-3p	4.574	-1.234	0.001

oha-miR-124-4-3p	4.934	-1.293	0.002
oha-miR-34c-5p	3.387	-1.380	0.025
ola-miR-124-3p	4.574	-1.234	0.001
pma-miR-124-3p	4.574	-1.234	0.001
pmi-miR-124-3p	4.574	-1.234	0.001
pol-miR-124-3p	4.574	-1.234	0.001
ppa-miR-124a	4.844	-0.794	0.001
ppc-miR-124	4.574	-1.234	0.001
ppy-miR-124	4.574	-1.234	0.001
ppy-miR-124a	4.844	-0.794	0.001
ppy-miR-146a	2.455	2.187	0.008
ppy-miR-34c-5p	3.493	1.219	0.023
ppy-miR-503	4.767	-4.230	0.049
prd-miR-124-3p	4.574	-1.234	0.001
ptr-miR-124a	4.844	-0.794	0.001
ptr-miR-146a	2.455	2.187	0.008
ptr-miR-503	4.767	-4.230	0.049
rno-miR-124-3p	4.574	-1.234	0.001
rno-miR-146a-5p	2.455	2.187	0.008
rno-miR-21-3p	1.429	1.818	0.010
rno-miR-322-5p	2.174	-2.821	0.045
rno-miR-34c-5p	3.493	1.219	0.023
sko-miR-124-3p	4.574	-1.234	0.001
sme-miR-124a-3p	4.458	-1.732	0.004
sme-miR-124b-3p	4.458	-1.732	0.004
sme-miR-124c-3p	4.574	-1.234	0.001
spu-miR-124	4.574	-1.234	0.001
ssc-miR-124a	4.574	-1.234	0.001
ssc-miR-146a-5p	2.455	2.187	0.008
ssc-miR-34c	3.493	1.219	0.023
str-miR-124-3p	4.574	-1.234	0.001
tca-miR-124-3p	4.574	-1.234	0.001
tch-miR-146a-5p	2.455	2.187	0.008
tch-miR-34c-5p	3.493	1.219	0.023
tgu-miR-124-3p	4.574	-1.234	0.001
tgu-miR-146c	2.455	2.187	0.008
tgu-miR-34b	3.493	1.219	0.023
tgu-miR-34c-5p	3.387	-1.380	0.025
tni-miR-124	4.574	-1.234	0.001
tur-miR-124-3p	4.574	-1.234	0.001
xbo-miR-124	4.574	-1.234	0.001
xtr-miR-124	4.844	-0.794	0.001
xtr-miR-34b	3.375	-1.478	0.019
**Down**			

aae-miR-184	-1.593	-0.003	0.001
aca-miR-184-3p	-1.552	1.521	0.002
aca-miR-200a-3p	-1.546	-2.375	0.016
aga-miR-184	-1.593	-0.003	0.001
ame-miR-184	-1.593	-0.003	0.001
api-miR-184a	-1.593	-0.003	0.001
bbe-miR-184-3p	-1.593	-0.003	0.001
bfl-miR-184-3p	-1.593	-0.003	0.001
bmo-miR-184-3p	-1.593	-0.003	0.001
bta-miR-184	-1.552	1.521	0.002
bta-miR-200a	-1.546	-2.375	0.016
bta-miR-3613a	-2.155	-2.365	0.003
bta-miR-369-3p	-1.007	2.041	0.007
bta-miR-376e	-1.501	-0.927	0.003
bta-miR-487a	-2.830	-3.755	0.033
bta-miR-6123	-2.370	-1.090	0.000
ccr-miR-184	-1.593	-0.003	0.001
ccr-miR-200a	-1.546	-2.375	0.016
cfa-miR-184	-1.552	1.521	0.002
cfa-miR-487a	-2.830	-3.755	0.033
cgr-miR-184	-1.552	1.521	0.002
cgr-miR-369-3p	-1.007	2.041	0.007
chi-miR-184	-1.552	1.521	0.002
chi-miR-200a	-1.546	-2.375	0.016
chi-miR-369-3p	-1.007	2.041	0.007
chi-miR-376e-3p	-1.501	-0.927	0.003
chi-miR-487a-3p	-2.830	-3.755	0.033
cin-miR-184	-1.593	-0.003	0.001
cqu-miR-184	-1.593	-0.003	0.001
cte-miR-184a	-1.593	-0.003	0.001
dan-miR-184-3p	-1.593	-0.003	0.001
der-miR-184-3p	-1.593	-0.003	0.001
dgr-miR-184-3p	-1.593	-0.003	0.001
dme-miR-184-3p	-1.593	-0.003	0.001
dmo-miR-184-3p	-1.593	-0.003	0.001
dpe-miR-184-3p	-1.593	-0.003	0.001
dps-miR-184	-1.593	-0.003	0.001
dre-miR-184	-1.593	-0.003	0.001
dre-miR-200a-3p	-1.546	-2.375	0.016
dse-miR-184-3p	-1.593	-0.003	0.001
dsi-miR-184-3p	-1.593	-0.003	0.001
dvi-miR-184-3p	-1.593	-0.003	0.001
dwi-miR-184-3p	-1.593	-0.003	0.001
dya-miR-184-3p	-1.593	-0.003	0.001

eca-miR-184	-1.552	1.521	0.002
eca-miR-200a	-1.546	-2.375	0.016
eca-miR-369-3p	-1.007	2.041	0.007
eca-miR-487a	-2.830	-3.755	0.033
efu-miR-200a	-1.546	-2.375	0.016
fru-miR-184	-1.593	-0.003	0.001
fru-miR-200a	-1.546	-2.375	0.016
gga-miR-184-3p	-1.552	1.521	0.002
gga-miR-200a-3p	-1.546	-2.375	0.016
ggo-miR-200a	-1.546	-2.375	0.016
ggo-miR-487a	-2.830	-3.755	0.033
hme-miR-184	-1.593	-0.003	0.001
hsa-miR-184	-1.552	1.521	0.002
hsa-miR-200a-3p	-1.546	-2.375	0.016
hsa-miR-3613-5p	-2.155	-2.365	0.003
hsa-miR-369-3p	-1.007	2.041	0.007
hsa-miR-487a-3p	-2.830	-3.755	0.033
ipu-miR-141	-1.546	-2.375	0.016
ipu-miR-184	-1.593	-0.003	0.001
ipu-miR-200a	-1.546	-2.375	0.016
isc-miR-184	-1.593	-0.003	0.001
lgi-miR-184	-1.593	-0.003	0.001
lva-miR-184-3p	-1.593	-0.003	0.001
mdo-miR-184-3p	-1.552	1.521	0.002
mdo-miR-200a-3p	-1.546	-2.375	0.016
Metazoa-novel-110-mature	-4.494	-4.124	0.023
Metazoa-novel-117-mature	-4.482	-4.113	0.041
Metazoa-novel-118-mature	-4.482	-4.113	0.041
Metazoa-novel-139-mature	-5.142	-3.822	0.017
Metazoa-novel-277-mature	-1.512	-2.519	0.026
Metazoa-novel-302-mature	-1.162	-1.862	0.040
mml-miR-184	-1.552	1.521	0.002
mml-miR-200a-3p	-1.546	-2.375	0.016
mml-miR-369-3p	-1.007	2.041	0.007
mml-miR-487a	-2.830	-3.755	0.033
mmu-miR-106a-5p	-2.709	-2.541	0.005
mmu-miR-184-3p	-1.552	1.521	0.002
mmu-miR-200a-3p	-1.546	-2.375	0.016

mmu-miR-369-3p	-1.007	2.041	0.007
mmu-miR-6402	-2.832	-3.773	0.011
mne-miR-184	-1.552	1.521	0.002
mse-miR-184	-1.593	-0.003	0.001
ngi-miR-184	-1.593	-0.003	0.001
nlo-miR-184	-1.593	-0.003	0.001
nvi-miR-184	-1.593	-0.003	0.001
oan-miR-200a-3p	-1.546	-2.375	0.016
oan-miR-23b-3p	-1.197	1.426	0.011
oar-miR-200a	-1.546	-2.375	0.016
oar-miR-369-3p	-1.007	2.041	0.007
oar-miR-376e-3p	-1.501	-0.927	0.003
oar-miR-487a-3p	-2.830	-3.755	0.033
odi-miR-1c	-1.384	0.144	0.027
oha-miR-200a	-1.546	-2.375	0.016
ola-miR-184-3p	-1.593	-0.003	0.001
ola-miR-200a	-1.546	-2.375	0.016
pmi-miR-184-3p	-1.593	-0.003	0.001
ppy-miR-184	-1.552	1.521	0.002
ppy-miR-200a	-1.546	-2.375	0.016
ppy-miR-369-3p	-1.007	2.041	0.007
ppy-miR-487a	-2.830	-3.755	0.033
ptr-miR-184	-1.552	1.521	0.002
ptr-miR-200a	-1.546	-2.375	0.016
ptr-miR-369	-1.007	2.041	0.007
ptr-miR-487a	-2.830	-3.755	0.033
rno-miR-184	-1.552	1.521	0.002
rno-miR-200a-3p	-1.546	-2.375	0.016
rno-miR-369-3p	-1.007	2.041	0.007
sha-miR-200a	-1.546	-2.375	0.016
sko-miR-184-3p	-1.593	-0.003	0.001
spu-miR-184	-1.593	-0.003	0.001
ssa-miR-200b-3p	-1.546	-2.375	0.016
ssc-miR-184	-1.552	1.521	0.002
ssc-miR-3613	-2.155	-2.365	0.003
ssc-miR-369	-1.007	2.041	0.007
str-miR-92-3p	-5.088	-3.823	0.002
tca-miR-184-3p	-1.593	-0.003	0.001
tca-miR-92a-3p	-1.799	-1.300	0.005
tch-miR-184	-1.552	1.521	0.002
tch-miR-200a-3p	-1.546	-2.375	0.016
tch-miR-369-3p	-1.007	2.041	0.007
tgu-miR-184	-1.552	1.521	0.002
tgu-miR-200a-3p	-1.546	-2.375	0.016

tni-miR-184	-1.593	-0.003	0.001
tni-miR-200a	-1.546	-2.375	0.016
tur-miR-184-3p	-1.593	-0.003	0.001
xtr-miR-184	-1.862	-0.723	0.000
xtr-miR-200a	-1.546	-2.375	0.016

[1]id：Mature miRNA id.

[2]logFoldChange：Differential multiple log2 conversion value.

[3]log2CPM：count-per-million log2 conversion value.

Table S15. Gene functions of 30 significantly enriched differential miRNAs targeting mRNA in the MHB group.

GO.ID	Term	Qntology	GeneRatio[1]	BgRatio[2]	*p*-Value
GO:0045944	positive regulation of transcription from RNA polymerase II promoter	biological process	202/3535	775/27054	0.000
GO:0000122	negative regulation of transcription from RNA polymerase II promoter	biological process	150/3535	533/27054	0.000
GO:0006355	regulation of transcription, DNA-templated	biological process	243/3535	1051/27054	0.000
GO:0045893	positive regulation of transcription, DNA-templated	biological process	114/3535	448/27054	0.000
GO:0008285	negative regulation of cell proliferation	biological process	72/3535	237/27054	0.000
GO:0006468	protein phosphorylation	biological process	131/3535	579/27054	0.000
GO:0007156	homophilic cell adhesion via plasma membrane adhesion molecules	biological process	36/3535	90/27054	0.000
GO:0045892	negative regulation of transcription, DNA-templated	biological process	90/3535	360/27054	0.000
GO:0001701	in utero embryonic development	biological process	58/3535	194/27054	0.000
GO:0007155	cell adhesion	biological process	65/3535	237/27054	0.000
GO:0005634	nucleus	cellular component	688/3535	3186/27054	0.000
GO:0005654	nucleoplasm	cellular component	498/3535	2246/27054	0.000
GO:0005829	cytosol	cellular component	473/3535	2176/27054	0.000
GO:0005737	cytoplasm	cellular component	526/3535	2612/27054	0.000
GO:0005886	plasma membrane	cellular component	441/3535	2418/27054	0.000
GO:0016020	membrane	cellular component	903/3535	5691/27054	0.000
GO:0014069	postsynaptic density	cellular component	39/3535	103/27054	0.000
GO:0005794	Golgi apparatus	cellular component	145/3535	682/27054	0.000

GO:0005623	cell	cellular component	130/3535	598/27054	0.000
GO:0043231	intracellular membrane-bounded organelle	cellular component	117/3535	525/27054	0.000
GO:0005515	protein binding	molecular function	691/3535	2880/27054	0.000
GO:0003677	DNA binding	molecular function	288/3535	1122/27054	0.000
GO:0005524	ATP binding	molecular function	312/3535	1328/27054	0.000
GO:0000978	RNA polymerase II core promoter proximal region sequence-specific DNA binding	molecular function	117/3535	380/27054	0.000
GO:0003700	transcription factor activity, sequence-specific DNA binding	molecular function	153/3535	573/27054	0.000
GO:0043565	sequence-specific DNA binding	molecular function	124/3535	444/27054	0.000
GO:0000166	nucleotide binding	molecular function	225/3535	1042/27054	0.000
GO:0046872	metal ion binding	molecular function	282/3535	1462/27054	0.000
GO:0042802	identical protein binding	molecular function	190/3535	897/27054	0.000
GO:0001227	transcriptional repressor activity, RNA polymerase II transcription regulatory region sequence-specific binding	molecular function	49/3535	148/27054	0.000

[1] GeneRatio: The number of target genes in this GO entry/the number of genes with GO annotations in the target gene.

[2] BgRatio: The number of genes in the GO entry of this entry/the number of genes with GO annotations in all genes.
